# Physics-Informed Identification of Spatial and Dynamic Responses of Dual-Element Pyroelectric Infrared Sensors Using Variable Circular-Aperture Excitation

**DOI:** 10.3390/s26216998

**Published:** 2026-11-05

**Authors:** Dang Bui Hai, Phat Nguyen Khac, Trung Van Duc, Quang Vu Van, Thang Vu Toan

**Affiliations:** 1 Precision Engineering & Smart Measurement Lab, School of Mechanical Engineering, Hanoi University of Science and Technology, Hanoi 100000, Vietnam; dangbh@utt.edu.vn (D.B.H.); nguyenphat14032004@gmail.com (P.N.K.); zinba31032004@gmail.com (T.V.D.); thang.vutoan@hust.edu.vn (T.V.T.); 2 University of Transportation Technology, Hanoi 100000, Vietnam

**Keywords:** pyroelectric infrared sensor, dual-element PIR, spatial-response calibration, system identification, frequency response, model structure selection, Fisher information, local identifiability, D-optimal experimental design, variable circular aperture

## Abstract

**Highlights:**

**What are the main findings?**

• The two-time-constant dynamic model commonly assumed for PIR modules misfits measured aperture-modulation data by up to 48%; a structure with two zeros at the origin and four poles reproduces it to 0.71%, the extra zero and pole coming from the AC-coupled conditioning stage.
• Evaluated at the identified operating point, the dynamic block is locally identifiable with cond(*J*_s_) = 75.8, and Fisher-based Cramér–Rao bounds agree with residual-bootstrap intervals to within ten per cent on every parameter.

**What are the implications of the main findings?**

• Ten greedily selected D-optimal excitation frequencies retain 61.6% of the information of a 40-point sweep, whereas an excitation grid chosen from an assumed operating point attains only 13.8%, so the excitation band must be fixed after—not before—structure identification.
• Referencing the analysis window to the excitation rather than to the sensor signal increases the Fisher determinant by a factor of 130 and removes actuator dynamics from the estimate, at no hardware cost.

**Abstract:**

This paper proposes a physics-informed identification framework for the spatial and dynamic responses of dual-element pyroelectric infrared (PIR) sensors using variable circular-aperture excitation. The dynamic half is carried through to experimental identification, while the spatial half is carried only to the reference-pose location, so the joint estimation is proposed rather than demonstrated. The spatial response is modeled as a bipolar differential kernel composed of a pose-dependent optical mapping, an optical envelope with a spatial scale factor, two smoothed rectangular sensing-element footprints, and a small antisymmetric residual correction. Sinusoidal modulation of the aperture radius leads to a complex first-harmonic frequency-domain model relating the measured response to aperture radius, modulation amplitude, pose setting, and excitation frequency. Rather than being assumed, the dynamic structure is identified from a 40-point frequency sweep spanning 0.0999 to 4.0453 Hz. A compact two-time-constant differentiating model leaves a root-mean-square log-magnitude error of 15.1%, with a maximum log-magnitude deviation of 48.4% (62% in relative amplitude). A structure with two zeros at the origin and four poles reproduces the same measurement to 0.71% root-mean-square. Its poles are an underdamped complex pair at 0.2126 Hz with damping ratio 0.899 and real poles at 0.5023 Hz and 2.9672 Hz. The additional zero and pole are tentatively attributed to the AC-coupled conditioning stage. Evaluated at this identified operating point, the scaled Jacobian of the dynamic block is full column rank with cond(*J*_s_) = 75.8, and Cramér–Rao bounds agree with residual-bootstrap intervals to within ten per cent on every parameter. A greedy D-optimal selection retains 61.6% of the information of the full sweep from ten frequencies, whereas the originally proposed excitation grid attains only 13.8% because it does not sample the band containing the identified pole pair. Restoring the excitation phase reference increases the Fisher determinant by a factor of 130. A structured estimation procedure is also formulated, in which the residual coefficients are updated through a regularized linear least-squares subproblem within a staged optimization exploiting the disparate conditioning of the spatial and dynamic blocks. It has not yet been executed on a joint aperture–pose–frequency data set.

## 1. Introduction

Pyroelectric infrared (PIR) sensors are widely used in low-cost and low-power thermal sensing systems because they provide simple hardware, passive operation, and practical robustness in motion-triggered applications such as occupancy monitoring, human activity detection, and embedded smart devices [1,2,3,4]. In many commercial modules, the pyroelectric sensing element is combined with a Fresnel lens or another non-imaging optical structure. As a result, the measured signal is governed not only by the intrinsic electro-thermal dynamics of the sensor and its conditioning circuit, but also by the effective spatial sensitivity pattern induced by the optics and by the relative alignment between the optical and sensing components [1,2,5,6]. Frequency-domain treatments of thermal diffusion have recently proved advantageous in infrared sensing [7], the principle on which Section 2.5 rests. For dual-element PIR modules, this spatial response is inherently differential and bipolar, enabling sensitivity to moving thermal targets while suppressing common-mode background radiation [2,5].

Recent studies have shown that the analog output of PIR sensors contains substantially richer information than simple binary motion triggering. PIR-based systems have been used for human movement monitoring and spatio-temporal data processing [1], indoor abnormal behavior detection [3], movement-direction inference [2], occupancy monitoring [4], and time-period or velocity-related estimation from PIR waveforms [8], and indoor positioning from PIR signals alone [9]. These works indicate that the waveform generated by a PIR module depends strongly on its spatio-temporal response, rather than only on the presence or absence of a thermal target. When a target moves across the field of view, the resulting signal is shaped by the underlying differential spatial kernel, the temporal dynamics of the pyroelectric element and electronics, and alignment imperfections between the sensing element and optical structure.

This motivates the need for a physically meaningful calibration of the spatial and dynamic response of PIR modules. Unlike imaging sensors, a PIR module does not directly provide a pixelized spatial map. Instead, it produces a one-dimensional electrical signal generated by radiative excitation integrated through the optics and filtered by the sensor’s dynamics. This makes the calibration problem intrinsically indirect. The difficulty is even more pronounced for dual-element PIR sensors, because the desired spatial response is not an unsigned sensitivity map but a signed differential kernel. In an ideal symmetric configuration, angular averaging may suppress the aperture-integrated differential response, even though the pointwise bipolar kernel remains nonzero and still determines the waveform generated by a moving thermal target [5,6].

A practical controlled excitation mechanism is variable circular-aperture excitation, in which the radiative input is modulated by changing the radius of a circular aperture. This excitation is experimentally attractive because it is simple to implement, compatible with compact optical setups, and produces a well-defined aperture-dependent input. However, it also introduces an important identifiability limitation: circular-aperture excitation does not directly reveal the full anisotropic spatial kernel. Instead, it probes the kernel only through aperture-integrated responses and, under small sinusoidal aperture modulation, through the derivative of the aperture response with respect to the aperture radius. Therefore, direct recovery of an unconstrained two-dimensional kernel from circular-aperture data is ill-posed.

For this reason, calibration should be formulated not as free-form spatial inversion, but as identification of a structured kernel family constrained by physical knowledge of the dual-element PIR module. In this work, the spatial response is modeled as a physics-informed differential kernel composed of a pose-dependent optical mapping, an optical envelope, and two smoothed rectangular sensing-element footprints. The rectangular dual-element geometry reflects the nominal sensing structure, while smoothing accounts for finite optical spot size, blur, and nonideal edge transitions. To retain flexibility without over-parameterizing the model, the envelope is allowed to vary through a spatial envelope scale factor, while directly measurable geometric quantities such as the element separation and element dimensions are fixed. A small antisymmetric residual correction is introduced at the differential-kernel level to accommodate optical imperfections, assembly tolerance, and residual asymmetry without destroying the dominant bipolar dual-element structure.

The aperture-response model is then linked to the measurable sensor output through a frequency-domain first-harmonic representation. Under sinusoidal modulation of the aperture radius, the first-order aperture response is proportional to the aperture-modulation amplitude and to the aperture-response derivative evaluated at the operating radius. This spatial input is then filtered by a low-order dynamic-response model of the PIR module. The resulting complex first-harmonic model relates the measured response to the aperture radius, modulation amplitude, pose setting, and excitation frequency. In the present formulation, a reduced parameter vector is used, including the unknown reference alignment pose, the spatial envelope scale factor, residual correction coefficients, and the dynamic parameters of the module.

Once the calibration model is written as a nonlinear parameter-estimation problem, local identifiability and experiment informativeness can be analyzed through the Jacobian and the Fisher information matrix [10,11,12,13,14]. These tools are widely used in nonlinear model calibration and optimal experimental design because they reveal weakly observable directions, parameter correlations, and the conditioning of the estimation problem [10,11,12,13,14]. In addition, because the residual coefficients enter the proposed model linearly for fixed nonlinear parameters, the estimation procedure can exploit the structure of separable nonlinear least squares, also known as variable projection [15,16]. This allows the residual coefficients to be updated through a regularized linear least-squares subproblem while the nonlinear physics and dynamic parameters are optimized iteratively.

The objective of this paper is to establish a practical physics-informed framework for identifying the spatial and dynamic responses of a dual-element PIR sensor from variable circular-aperture data. Rather than attempting to reconstruct an arbitrary two-dimensional response map, the proposed approach identifies a reduced but physically interpretable model. The analysis focuses on whether the selected aperture radii, pose settings, and excitation frequencies provide sufficient information for the reduced model, and on which parameter directions remain weakly observable. The broader issues of robust design under larger model uncertainty and uncertainty propagation to moving-target prediction are left as natural extensions of the present framework.

The main contributions of this work are summarized as follows.

**First**, we develop a reduced physics-informed spatial–dynamic model for a dual-element PIR sensor. The model preserves the bipolar differential sensing mechanism through a pose-dependent optical mapping, a spatial envelope with a scale factor, and two smoothed rectangular sensing-element footprints. A small antisymmetric residual correction is included to represent practical nonidealities while keeping the dominant dual-element structure explicit.

**Second**, we formulate a variable circular-aperture calibration model in the frequency domain. By applying small sinusoidal aperture modulation, the aperture-integrated spatial response is converted into a complex first-harmonic model that links the measured signal to aperture radius, modulation amplitude, pose setting, and excitation frequency. This formulation provides a practical route from controlled aperture excitation to spatial–dynamic parameter identification.

**Third**, we evaluate local identifiability and propose a structured estimation procedure for the reduced model. The scaled Jacobian and Fisher information matrix are used to examine rank, conditioning, parameter sensitivity, and weakly observable directions. The residual coefficients are handled through a regularized linear least-squares subproblem within a staged optimization procedure, improving numerical stability while avoiding excessive freedom in the residual correction.

**Fourth**, we identify the model experimentally rather than assuming it. A 40-point frequency sweep establishes the dynamic structure and its operating point, a two-stage design of experiments locates the reference pose, and the Fisher analysis is evaluated at the identified point so that the resulting D-optimal design describes the physical module.

The remainder of this paper is organized as follows. Section 2 presents the physics-informed dual-element spatial model and derives the circular-aperture first-harmonic frequency-domain identification model. Section 3 reports the experimental characterization of the dynamic response. Section 4 analyzes local identifiability using the scaled Jacobian and Fisher information matrix and derives the D-optimal experiment design. Section 5 reports the experimental identification of the spatial response. Section 6 describes the structured estimation procedure. Section 7 discusses the implications, limitations, and possible extensions of the framework, and Section 8 concludes the paper.

## 2. Physics-Informed Spatial–Dynamic-Response Model for Dual-Element PIR Sensors

This section formulates the spatial–dynamic-response model used for aperture-based calibration of a dual-element PIR module. The objective is not to reconstruct an arbitrary two-dimensional spatial-response map, but to identify a reduced physics-informed model that preserves the bipolar differential sensing structure and remains observable from circular-aperture excitation.

### 2.1. Coordinate Mapping and Reference Pose

**Figure 1:**
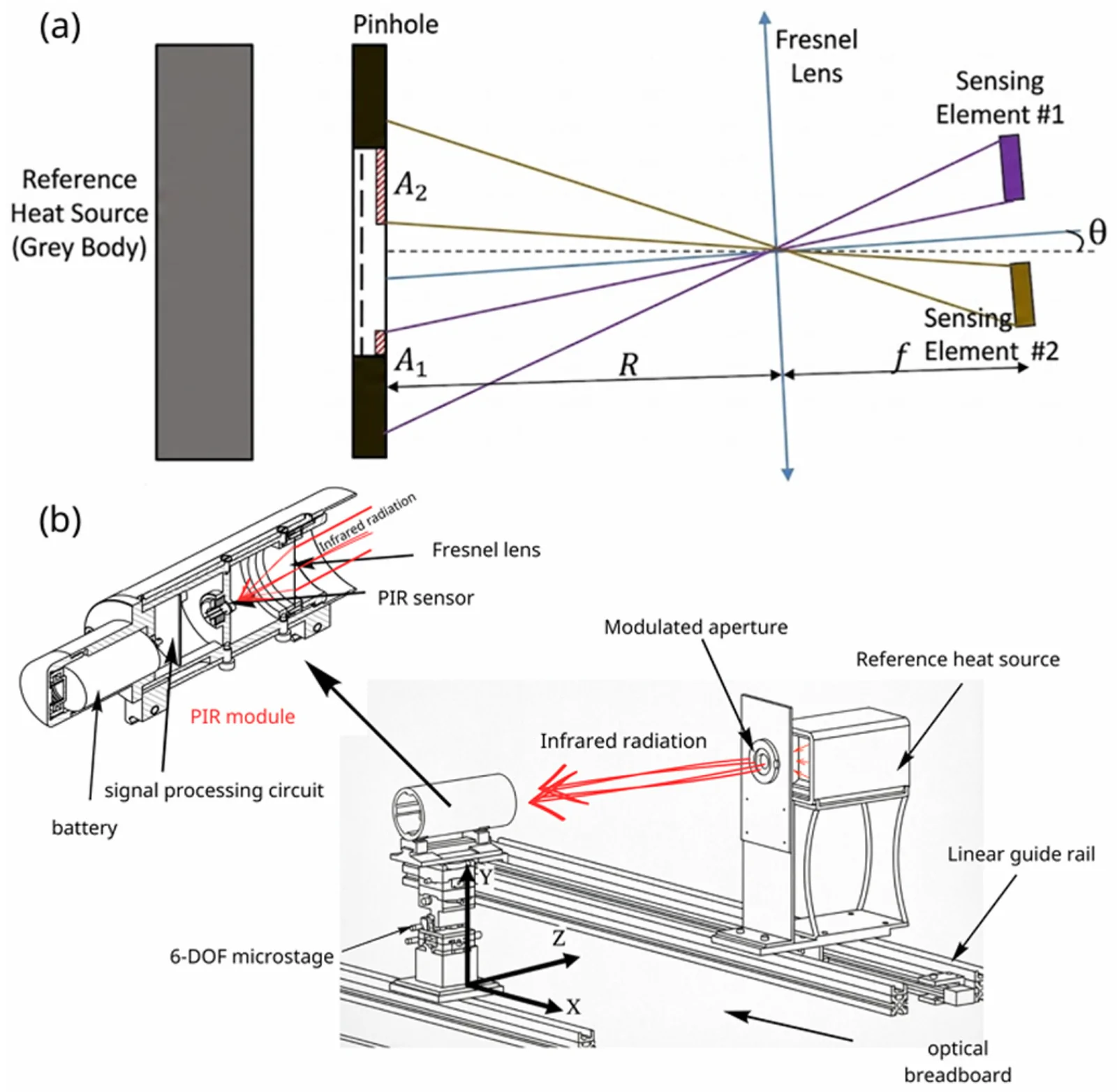
Variable circular-aperture excitation setup. (**a**) Optical geometry at the sensing-element plane: radiation through the instantaneous open areas A_1_ and A_2_ crosses a distance R to the Fresnel lens and is imaged at focal distance f onto elements #1 and #2; θ is the residual optical–mechanical tilt. (**b**) Working principle and main components of the bench, with a cut-away view of the PIR module.

The experimental configuration to which the model refers is shown in Figure 1. Let the aperture or source plane be described by the physical coordinates

(1) $$q_{s}={\left[\;X_{s},\;Y_{s},\;Z_{s}\;\right]}^{T},$$

where *Z_s_* is the nominal distance from the aperture plane to the PIR module. The PIR module is supposed to be placed at coordinates (0, 0, 0). The known experimental pose is denoted by

(2) $$p={\left[\;\alpha_{x},\;\alpha_{y},\;\alpha_{z}\;\right]}^{T},$$

where *α_x_*, *α*_y_, *α*_z_ are small rotations. The pose vector carries the three angular degrees of freedom only. A lateral translation is not separate because, to first order in Equations (7) and (8), a displacement at the stand-off gives the same sensor-plane shift as a tilt of that displacement divided by the stand-off, which the translation–tilt coupling of Section 5.3 expresses. The unknown perfectly aligned reference pose is

(3) $$p^{*}={\left[\;\alpha_{x}^{*},\;\alpha_{y}^{*},\;\alpha_{z}^{*}\;\right]}^{T}.$$

The reference pose p* represents the hypothetical pose at which the optical axis, aperture center, and dual-element sensing structure are perfectly aligned. In practice, this pose is not directly measurable because commercial PIR modules include assembly tolerances and lens–sensor centering errors. Therefore, p* is treated as an unknown calibration parameter, while the experimentally imposed poses p are known inputs. Identifiability in principle and component-wise resolution differ. The kernel of Equation (15) is odd about the optical axis, so the response of Equation (23) vanishes when the aperture axis meets its axis of antisymmetry, making a null a signature of alignment. It fails component-wise for a scalar response at one aperture radius, given the translation–tilt coupling of Section 4.3 and Section 5.3; it needs a varying radius and the complex response of Equation (36).

The relative pose deviation is defined as

(4) $$\Delta p=p-p^{*}={\left[\;\Delta \alpha_{x},\;\Delta \alpha_{y},\;\Delta \alpha_{z}\;\right]}^{T}.$$

For a point on the aperture plane, the corresponding coordinate in the PIR reference frame is modeled as

(5) $$q_{p}={\left[\;X_{p},\;Y_{p},\;Z_{p}\;\right]}^{T}=R\left(\Delta \alpha_{x},\;\Delta \alpha_{y},\;\Delta \alpha_{z}\right)\;{\left[\;X_{s},\;Y_{s},\;Z_{s}\;\right]}^{T},$$

where

(6) *R*(Δ*α_x_*, Δ*α_y_*, Δ*α_z_*) = *R_z_*(Δ*α_z_*) *R_y_*(Δ*α_y_*) *R_x_*(Δ*α_x_*).

The corresponding angular coordinates viewed by the PIR optics are approximated by

(7) $$u=\frac{X_{p}}{Z_{p}},\;\;v=\frac{Y_{p}}{Z_{p}}.$$

The effective coordinates on the sensor plane are then written as

(8) *x* = *f*_eff_ *u*,  *y* = *f*_eff_ *v*,

where *f*_eff_ is the effective focal or optical mapping scale of the PIR module. The coordinates (x, y) are therefore determined by the pose-dependent mapping from the aperture plane to the sensor plane. This mapping is a reduced local approximation of the non-imaging optical transformation and is used to capture the dominant effects of translation and angular misalignment, which are known to be important in dual-element PIR centering-error studies [5,6].

### 2.2. Physics-Based Dual-Element Spatial Kernel

The dual-element PIR sensor is modeled as two smoothed rectangular sensing elements with opposite signs in the differential response. This reflects the common physical structure of dual-element PIR modules, where two pyroelectric elements are connected in a differential configuration to enhance sensitivity to moving thermal targets while suppressing common-mode radiation [1,2,5].

Let the nominal element centers on the sensor plane be

(9) *r*_1_ = (−*d*/2, 0),  *r*_2_ = (+*d*/2, 0),

where d is the center-to-center separation between the two sensing elements. The effective element width and height are denoted by w and h, respectively. A smoothed rectangular footprint centered at (*c_x_*, c_y_) is defined as

(10) *S*(*x*, *y*; *c_x_*, *c_y_*, *w*, *h*, *σ_x_*, *σ_y_*) = *F_x_*(*x*) *F_y_*(*y*),

where

(11) $$F_{x}\left(x\right)=\frac{1}{2}\left[\mathrm{erf}\left(\frac{x-c_{x}+w/2}{\sqrt{2}\;\sigma_{x}}\right)-\mathrm{erf}\left(\frac{x-c_{x}-w/2}{\sqrt{2}\;\sigma_{x}}\right)\right],$$

and

(12) $$F_{y}\left(y\right)=\frac{1}{2}\left[\mathrm{erf}\left(\frac{y-c_{y}+h/2}{\sqrt{2}\;\sigma_{y}}\right)-\mathrm{erf}\left(\frac{y-c_{y}-h/2}{\sqrt{2}\;\sigma_{y}}\right)\right].$$

The two element footprints are

(13) *S*_1_(*x*, *y*) = *S*(*x*, *y*; −*d*/2, 0, *w*, *h*, *σ_x_*, *σ_y_*), *S*_2_(*x*, *y*) = *S*(*x*, *y*; +*d*/2, 0, *w*, *h*, *σ_x_*, *σ_y_*).

The smoothed footprint S(x, y) is used as a differentiable approximation of the rectangular sensing area of each pyroelectric element. The error-function form can be interpreted as an ideal rectangular element convolved with a Gaussian blur, accounting for finite optical spot size and nonideal edge transitions. This avoids discontinuous spatial derivatives and improves numerical stability in the subsequent Jacobian- and Fisher-based analysis [10,11,12]. The smoothing parameters *σ_x_* and *σ*_y_ account for finite optical spot size, blur, and nonideal edge transitions. This also makes the spatial model differentiable and suitable for numerical sensitivity analysis.

The optical envelope E(x, y; *k_s_*) describes the finite spatial support and gradual sensitivity attenuation of the PIR optical system. The scale factor *k_s_* uniformly expands or contracts the nominal envelope widths, allowing the effective sensing region to be calibrated without introducing separate free envelope parameters. The optical envelope is modeled as

(14) $$E\left(x,\;y;\;k_{s}\right)=\exp\left\{-\frac{1}{2}\left[{\left(\frac{x}{k_{s}s_{x0}}\right)}^{2}+{\left(\frac{y}{k_{s}s_{y0}}\right)}^{2}\right]\right\},$$

where s_x0_ and s_y0_ are nominal envelope widths, and *k_s_* is the spatial envelope scale factor. The parameter *k_s_* allows the effective spatial support of the kernel to expand or contract without introducing separate envelope-width parameters.

The physics-based dual-element differential kernel is then

(15) *W*_phys_(*x*, *y*; *k_s_*) = *E*(*x*, *y*; *k_s_*)[*S*_1_(*x*, *y*) − *S*_2_(*x*, *y*)].

This construction preserves the bipolar response structure of a dual-element PIR sensor. For the module used in this work, the two elements measure 2 × 1 mm with a 1 mm gap, giving d = 2 mm, and the Fresnel lens has a diameter of 50 mm and a focal length of 50.9 mm over the 5–12 µm waveband.

### 2.3. Antisymmetric Residual Correction

The physics-based kernel cannot represent all practical nonidealities of a commercial PIR module. Optical imperfections, assembly tolerances, slight element asymmetries, and lens–sensor mismatch may introduce deviations from the nominal differential kernel [5,6]. Therefore, a small residual correction is introduced at the differential-kernel level:

(16) *W*_total_(*x*, *y*) = *W*_phys_(*x*, *y*; *k_s_*) + *W*_res_(*x*, *y*; *c*).

To preserve the differential nature of the dual-element response, the residual term is represented using antisymmetric basis functions. Define

(17) $$\tilde{x}=\frac{x}{s_{r}},\;\;\tilde{y}=\frac{y}{s_{r}},\;\;{\tilde{r}}^{2}={\tilde{x}}^{2}+{\tilde{y}}^{2},\;\;G\left(\tilde{x},\;\tilde{y}\right)=\exp\left(-{\tilde{r}}^{2}\right),$$

where *s_r_* is a fixed residual support scale. The residual basis functions are chosen to be odd under reflection of the lateral coordinate, the symmetry generated by the differential readout of the element pair:

(18) $$\psi_{1}=\tilde{x}\;\tilde{y}\;G,\;\;\psi_{2}=\left({\tilde{x}}^{3}-3\;\tilde{x}\;{\tilde{y}}^{2}\right)G.$$

Both functions change sign under reflection about the vertical axis of the sensor plane,

(19) $$\psi_{m}\left(-\tilde{x},\;\tilde{y}\right)=-\psi_{m}\left(\tilde{x},\;\tilde{y}\right),\;\;m=1,\;2,$$

so each basis function reverses sign when the two sensing elements are interchanged, the symmetry the differential readout imposes. The constraining group is this reflection, not point inversion: under inversion of both coordinates the second basis function is odd, the first even. In polar coordinates they carry the angular factors sin 2φ and cos 3φ, so both vanish over an aperture centered on the axis; off-center they enter Equation (28) with different weights, making the coefficients separately observable. The residual kernel is defined as

(20) $$W_{\mathrm{res}}\left(x,\;y;\;c\right)=E\left(x,\;y;\;k_{s}\right)\sum_{m=1}^{2}c_{m}\;\psi_{m}\left(\tilde{x},\;\tilde{y}\right).$$

**Figure 2:**
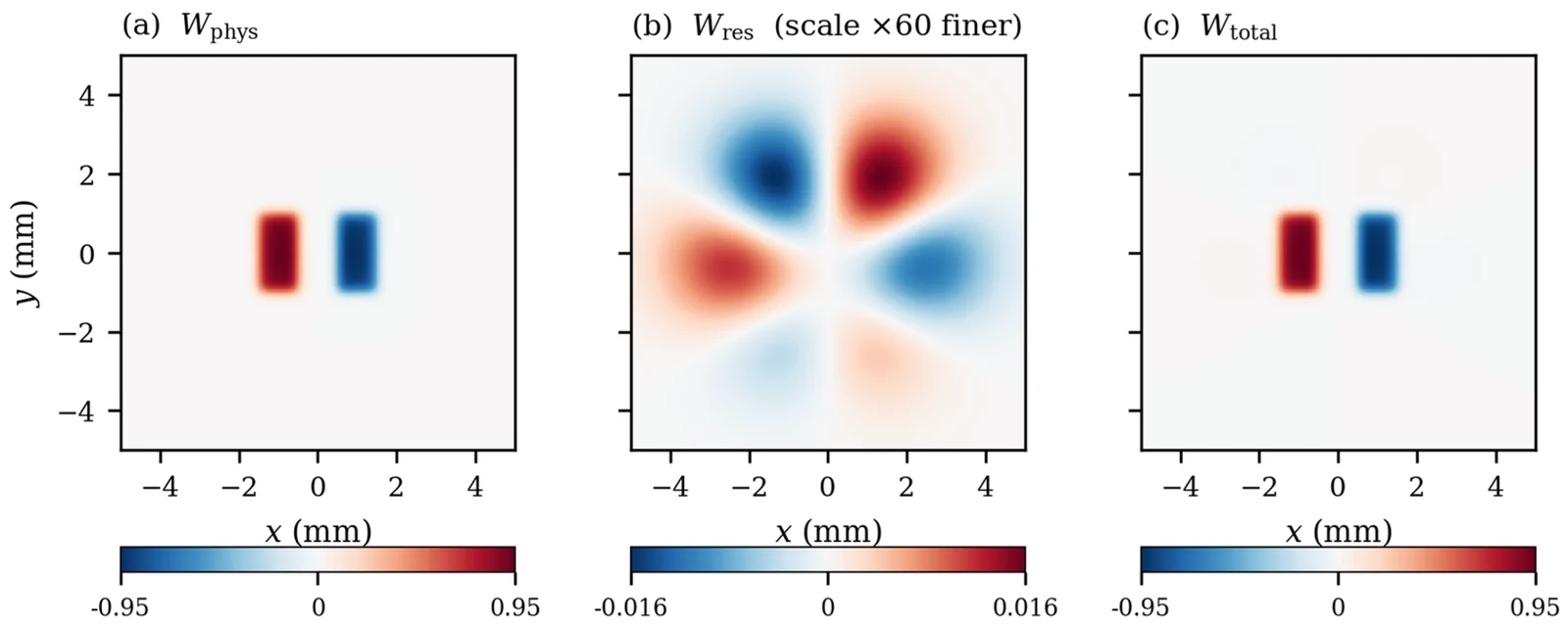
Physics-informed spatial kernel of Equations (15), (20) and (21), evaluated on the sensor plane. This panel set is a computed illustration of the model structure; it is not measured data and not a calibrated result. Geometric quantities are those of the module used in this work: element footprint 2 × 1 mm, center-to-center separation d = 2 mm, Fresnel lens of 50 mm diameter and 50.9 mm focal length. The remaining quantities are fixed at representative values for display only: *σ_x_* = *σ*_y_ = 0.15 mm, s_x0_ = 3.0 mm, s_y0_ = 2.5 mm, k_s_ = 1, s_r_ = 2.5 mm, *c*_1_ = 0.06, *c*_2_ = −0.04. (**a**) Physics-based bipolar kernel W_phys_. (**b**) Antisymmetric residual correction W_res_, plotted on a color scale sixty times finer because it is two orders of magnitude smaller than (**a**). (**c**) Total kernel W_total_.

Figure 2 illustrates the three components of the kernel for the module geometry used in this work. The multiplication by the same optical envelope E(x, y; *k_s_*) limits the residual correction to the optical support of the PIR module and prevents it from replacing the dominant physics-based kernel. The total spatial kernel used in the calibration model is therefore

(21) *W*_total_(*x*, *y*; p^*^, *k_s_*, *c*) = *W*_phys_(*x*, *y*; *k_s_*) + *W*_res_(*x*, *y*; *c*),

where (x, y) are determined by the pose-dependent mapping from the aperture plane to the sensor plane.

### 2.4. Circular-Aperture Response

Let the circular aperture of radius a be centered on the source plane. The illuminated region is

(22) $$A\left(a\right)=\left\{\left(X_{s},\;Y_{s}\right):X_{s}^{2}+Y_{s}^{2}\leq a^{2}\right\}.$$

The cumulative aperture response is defined as

(23) $$g\left(a,\;p;\;\theta \right)=\iint_{A\left(a\right)}W_{\mathrm{total}}\left(x\left(X_{s},\;Y_{s},\;Z_{0};\;p,\;p^{*}\right),\right.$$

(*y*(*X_s_*, *Y_s_*, *Z*_0_; *p*, p^*^); *θ*)*dX_s_* *dY_s_*.

This expression shows that circular-aperture excitation does not observe the spatial kernel pointwise. Instead, it probes the kernel through an aperture-integrated transform. This is the reason why a structured physics-informed kernel is used instead of a free-form spatial inversion.

For harmonic identification, the important quantity is the derivative of the aperture response with respect to the aperture radius. Using the boundary form of the area derivative,

(24) $$\frac{\partial g}{\partial a}\left(a,\;p;\;\theta \right)=\int_{0}^{2\pi }W_{\mathrm{total}}\left(x\left(\varphi \right),\;y\left(\varphi \right);\;\theta \right)a\;\mathrm{d\varphi},$$

where the circular boundary is parameterized by

(25) *X_s_*(*φ*) = *a*cos*φ*,  *Y_s_*(*φ*) = *a*sin*φ*,  *Z_s_* = *Z*_0_ = const.

The sensor-plane boundary coordinates are

(26) (*x*(*φ*), *y*(*φ*)) = *T*(*p*, p^*^)(*a*cos*φ*, *a*sin*φ*, *Z*_0_),

where T(p, p*) denotes the pose-dependent source-to-sensor mapping. Using W_total_ = W_phys_ + W_res_, the aperture derivative can be decomposed as

(27) $$\frac{\partial g}{\partial a}=\frac{\partial g_{\mathrm{phys}}}{\partial a}+\frac{\partial g_{\mathrm{res}}}{\partial a}.$$

Because the residual kernel is linear in the coefficients *c_m_*, the residual part of the aperture derivative is also linear in *c_m_*:

(28) $$\frac{\partial g_{\mathrm{res}}}{\partial a}=\sum_{m=1}^{2}c_{m}\;b_{m}\left(a,\;p;\;\eta \right),$$

where

(29) $$b_{m}\left(a,\;p;\;\eta \right)=\int_{0}^{2\pi }E\left(x\left(\varphi \right),\;y\left(\varphi \right);\;k_{s}\right)\psi_{m}\left(x\left(\varphi \right),\;y\left(\varphi \right)\right)a\;\mathrm{d\varphi}.$$

Here *η* denotes the nonlinear parameter block excluding the linear residual coefficients. This separable structure motivates the variable-projection estimation strategy used later in the paper [15,16].

### 2.5. First-Harmonic Frequency-Domain Model

The aperture radius is modulated sinusoidally around an operating radius *a*_0_:

(30) *a*(*t*) = *a*_0_ + *A_m_*sin(*Ωt*),

where *A_m_* is the modulation amplitude and *Ω* = 2πf is the angular excitation frequency. For small modulation amplitudes, a first-order Taylor expansion gives

(31) $$g\left(a\left(t\right),\;p;\;\theta \right)\approx g\left(a_{0},\;p;\;\theta \right)+A_{m}\frac{\partial g}{\partial a}\left(a_{0},\;p;\;\theta \right)\sin\left(\Omega |t\right).$$

Thus, the first-harmonic spatial input has amplitude *A_m_* (∂g/∂a)(*a*_0_, p; *θ*). In the present experimental design, the modulation amplitude is selected as *A_m_*/*a*_0_ = 0.1, which supports the use of the first-order aperture model.

The full complex first-harmonic response of the PIR module is denoted by *Y_i_*. Using the dynamic transfer function H(jΩ), the full harmonic response for experiment i is

(32) $$Y_{i}=A_{m,i}\;H\left(j\Omega_{i}\right)\;\frac{\partial g}{\partial a}\left(a_{0,i},\;p_{i};\;\theta \right).$$

Section 2 adopts, as a working hypothesis, the compact two-time-constant differentiating form

(33) $$H\left(j|\Omega \right)=\frac{K\;j\Omega }{\left(1+j\Omega \tau_{1}\right)\left(1+j\Omega \tau_{2}\right)},$$

in which the zero at the origin represents the pyroelectric current response to the rate of change in the absorbed radiation, and the two poles represent the thermal and electrical relaxation of the sensing element. In this work, the objective is to identify the time constants of the module and the spatial-response parameters, rather than the absolute gain K. Since *jΩ_i_* is known for each excitation frequency and K acts as a global gain factor, the response is normalized as

(34) $${\tilde{Y}}_{i}=\frac{Y_{i}}{K\;j\Omega_{i}}.$$

Therefore, the normalized identification model becomes

(35) $${\tilde{Y}}_{i}\left(\theta \right)=A_{m,i}\;H_{n}\left(j\Omega_{i}\right)\;\frac{\partial g}{\partial a}\left(a_{0,i},\;p_{i};\;\theta \right),$$

where *H_n_* denotes the normalized dynamic response obtained after removing the known differentiating factor and the global gain. Equivalently,

(36) $${\tilde{Y}}_{i}\left(\theta \right)=A_{m,i}\;H_{n}\left(\Omega_{i}\right)\int_{0}^{2\pi }W_{\mathrm{total}}\left(\xi_{i}\left(\varphi \right),\;\eta_{i}\left(\varphi \right);\;\theta \right)a_{0,i}\;\mathrm{d\varphi}.$$

This equation is the reduced frequency-domain identification model used in the subsequent identifiability analysis. For notational consistency, Ỹ_i_ denotes the normalized first-harmonic response throughout the remainder of the paper. The specific form of *H_n_* is not taken for granted: Section 3 tests the hypothesis in Equation (33) against measured data and replaces it where necessary.

### 2.6. Reduced Parameter Vector

The unknown parameter vector is

(37) $$\theta ={\left[p^{*T},\;k_{s},\;c_{1},\;c_{2},\;\theta_{\mathrm{dyn}}^{T}\right]}^{T}={\left[\alpha_{x}^{*},\;\alpha_{y}^{*},\;\alpha_{z}^{*},\;k_{s},\;c_{1},\;c_{2},\;\theta_{\mathrm{dyn}}^{T}\right]}^{T},$$

where *θ*_dyn_ collects the dynamic parameters of *H_n_*. Under the working hypothesis of Equation (33), *θ*_dyn_ = [*τ*_1_, *τ*_2_]^T^ and the model contains eight parameters. The experimental design variables are treated as known inputs:

(38) *D_i_* = (*a*_0,_*_i_*, *A_m_*_,_*_i_*, *f_i_*, *p_i_*).

In the implementation used in this work, *a*_0_ = [3.3, 6.6, 9.9] mm and *A_m_* = 0.1 *a*_0_, while the excitation frequencies cover the range specified by the experiment design of Section 4. Thus, the model combines a reduced spatial parameterization with a compact dynamic response, leading to an 8-parameter spatial–dynamic identification problem under the working hypothesis, and to a 10-parameter problem once the dynamic structure identified in Section 3 is adopted.

## 3. Experimental Characterization of the Dynamic Response

Section 2 introduced the dynamic response as a compact two-time-constant differentiating transfer function. That form is physically motivated but remains a hypothesis. Because the Fisher information matrix of a nonlinear model is evaluated at a nominal operating point, every identifiability and design conclusion in Section 4 inherits both the structure and the operating point assumed for *H_n_*. A structurally inadequate model therefore does not merely degrade the fit; it displaces the entire information landscape on which the design is built. The dynamic structure is consequently established experimentally first.

This section reports a 40-point frequency sweep, tests the two-time-constant hypothesis against it, selects a structurally adequate replacement using information criteria and residual diagnostics, and quantifies parameter uncertainty by residual bootstrap and profile likelihood. It closes by examining the reliability of the measured phase, which constrains what the present data can identify.

### 3.1. Measurement Configuration and Signal Processing

**Figure 3:**
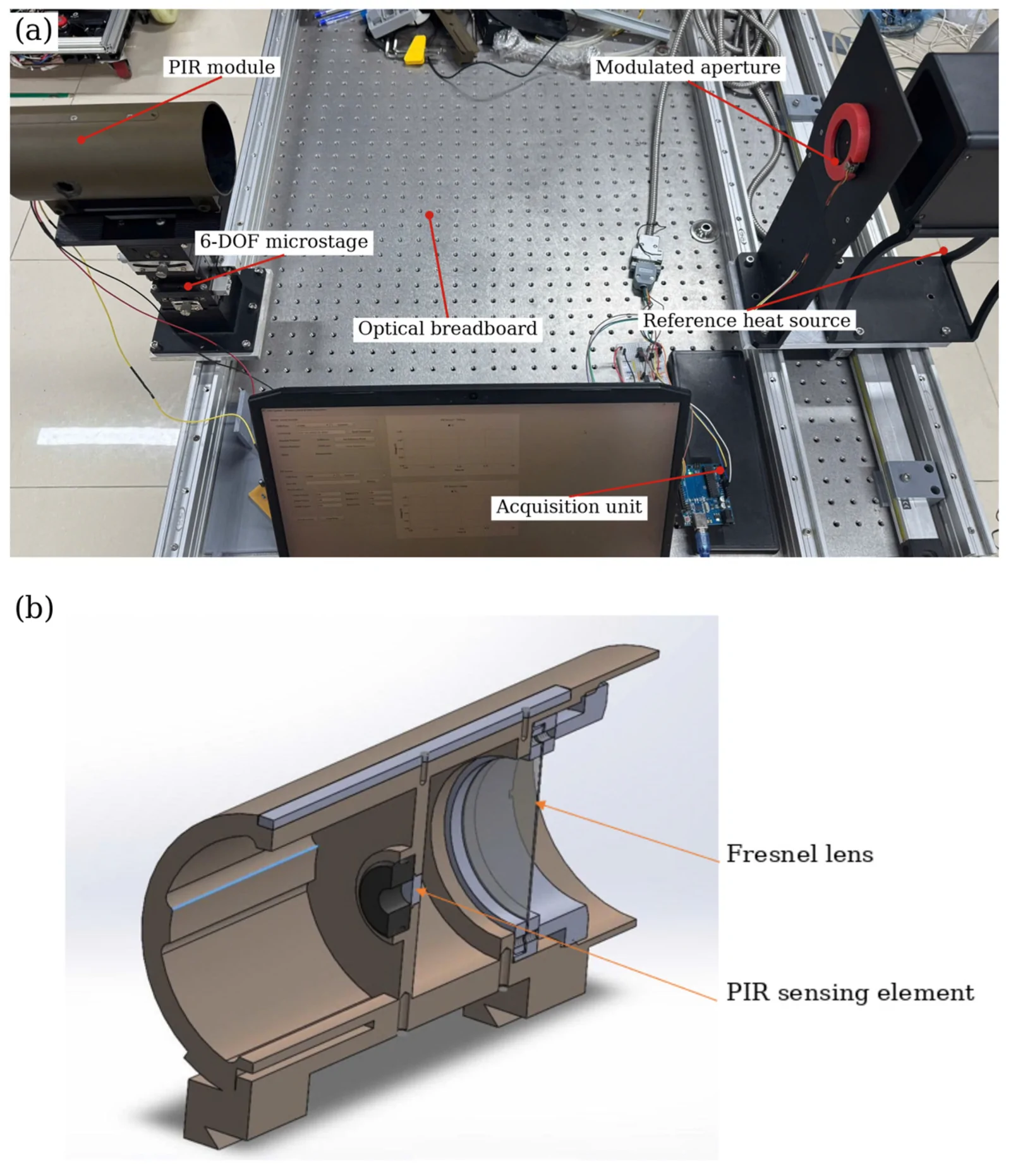
Experimental apparatus used for the frequency sweep. (**a**) Optical bench: the PIR module is mounted on the six-degree-of-freedom microstage on the left, the modulated aperture and the reference heat source are on the right, and the acquisition unit drives the aperture actuator and samples the sensing channel. (**b**) Cross-section of the PIR module, showing the Fresnel lens and the dual-element pyroelectric detector at its focal plane.

The apparatus is shown in Figure 3. The aperture radius was modulated sinusoidally about a fixed operating radius with a fixed modulation amplitude, and the excitation frequency was swept over 40 settings while the pose was held at the nominal configuration. The PIR module output was recorded on the sensing channel, and the excitation command was generated by a stepper-driven variable aperture whose commanded period is expressed in units of the driver timer tick, so that the nominal excitation frequency follows

(39) $$f=\frac{1200}{T_{\mathrm{tick}}},$$

with T_tick_ the commanded period in ticks. The sampling rate was recovered from the ratio of the number of samples in each analysis window to the window duration and was found to be 500.09 ± 0.06 Hz across all 40 records, confirming a stable acquisition clock.

For each record, the mean was removed, and a rectangular window spanning exactly 15 excitation cycles was applied. Coherent windowing places the excitation on a DFT bin, suppressing leakage of the fundamental without an amplitude-distorting taper [17]. The measured first-harmonic response is the complex DFT coefficient at that bin, denoted by *Y_i_^m^* to distinguish it from the normalized Ỹ_i_ of Equation (35). Because the cycle count rather than the window duration is held constant, the relative resolution *Δ*f/f = 1/15 is uniform.

The configuration is summarized in Table 1. Two of its properties matter here. First, *a*_0_, *A_m_* and the pose were all held constant, so the aperture-derivative factor in Equation (32) is a constant of the sweep and the measured magnitude is proportional to |H(jΩ)| alone: the sweep isolates the dynamic response and is an uncontaminated test of its structure. Second, and for the same reason, it carries no information about the spatial parameters, which must be excited by varying *a*_0_ and p.

### 3.2. Measured First-Harmonic Frequency Response

**Figure 4:**
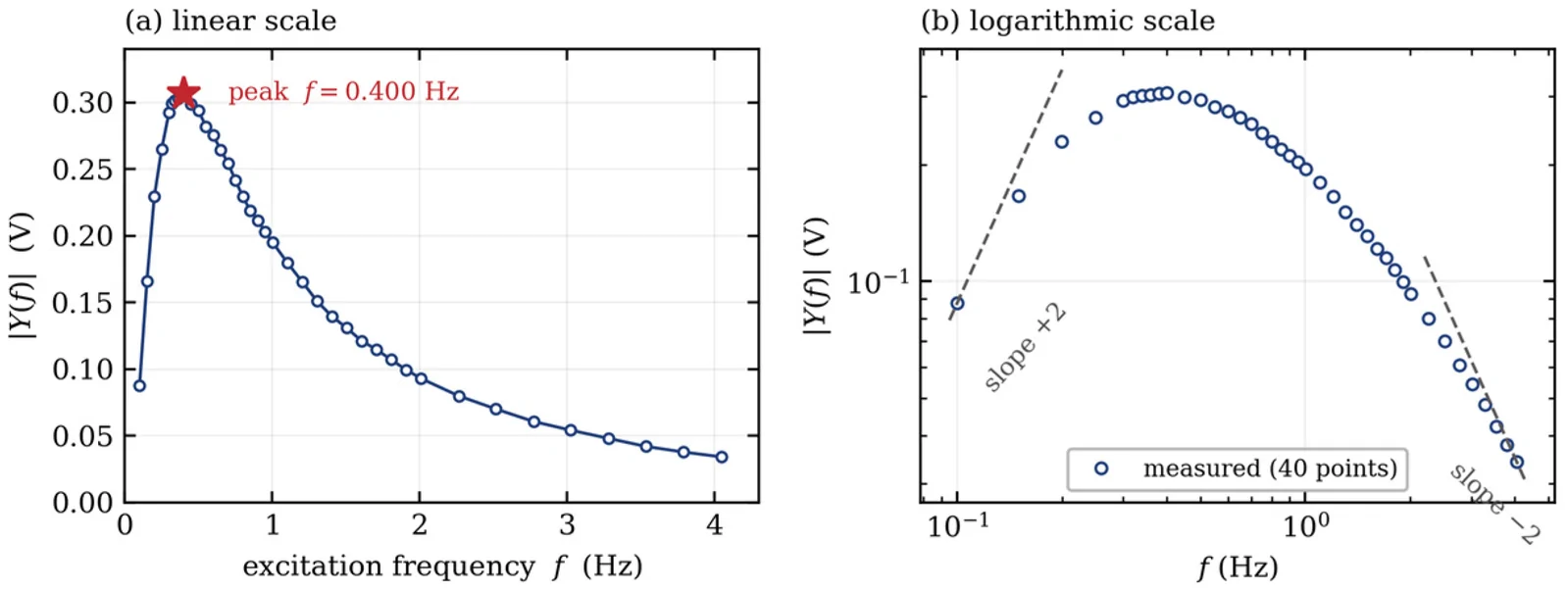
Measured first-harmonic magnitude of the PIR response under sinusoidal aperture modulation, 40 excitation frequencies. (**a**) Linear axes, showing the band-pass character and the response maximum at 0.3996 Hz. (**b**) Logarithmic axes; the dashed guides are the theoretical asymptotic slopes of +2 and −2 of the model M_2_ selected in Section 3.4, not fits to the data; the measured local slopes over the extremes of the sweep are +1.57 and −1.54 and are still steepening at the band edges.

The measured magnitude |*Y_i_^m^*| is shown in Figure 4 on linear and logarithmic axes. The response is unambiguously band-pass. It rises steeply from 0.0878 V at 0.0999 Hz, attains a maximum of 0.3068 V at 0.3996 Hz, and falls monotonically to 0.0343 V at 4.0453 Hz, giving a dynamic range of 8.95:1 across the sweep. The curve is smooth and free of outliers, and the point-to-point scatter is small compared with the systematic curvature, which indicates good measurement repeatability.

Figure 4b exposes the feature that determines the structure. Local straight-line fits at the extremes give a low-frequency log–log slope of +1.57 over the two lowest frequencies and +1.23 over the four lowest, and −1.54 over the six highest. Both asymptotes are steeper than unity and still steepening at the band edges, whereas Equation (33), with one zero at the origin and two poles, constrains them to exactly +1 and −1. The measured response is thus structurally incompatible with the assumed model at both ends, independently of any parameter values.

### 3.3. Adequacy of the Two-Time-Constant Structure

**Figure 5:**
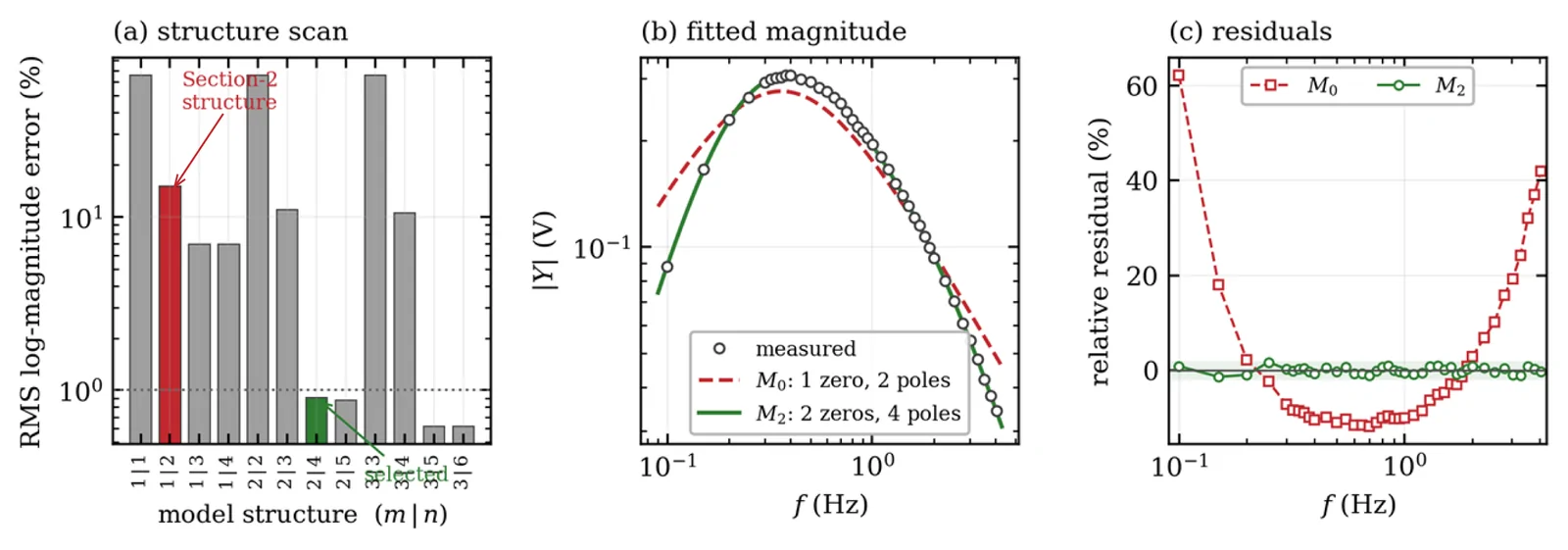
Structural discrimination of the dynamic model. (**a**) Root-mean-square log-magnitude error for each candidate structure; the structure assumed in Section 2 is highlighted in red and the selected structure in green. (**b**) Measured magnitude with the fitted Section 2 structure *M*_0_ and the selected structure *M*_2_. (**c**) Relative amplitude residuals of the two models, 100 × (|H| − |*Y^m^*|)/|*Y^m^*|; note that this is the relative scale, so the peak of 62% for M_0_ corresponds to the 48.40% log-magnitude deviation listed in Table 2. The shaded band marks ±2%.

To quantify this, Equation (33) was fitted directly to the measured magnitudes in log-magnitude space—the appropriate metric for multiplicative error over nearly a decade of amplitude [17,18]—from 100 randomized starts. The best fit gives K = 0.2444, *τ*_1_ = *τ*_2_ = 0.4427 s, with a root-mean-square log-magnitude error of 15.09% and a maximum log-magnitude deviation of 48.40% at the lowest frequency. Throughout this paper, a log-magnitude error of *δ* per cent denotes 100 × |ln|H| − ln|*Y^m^*||; since ln(1 + x) ≈ x only for small x, 48.40% corresponds to a relative amplitude error of exp(0.4840) − 1 = 62.3%, the quantity plotted in Figure 5c.

**Table 2:** Structural model scan on the measured magnitude response. m is the number of zeros at the origin, n the number of real poles, and k the number of free parameters including the gain. Structures with n ≤ m are omitted: all leave a 65.85% error because the model cannot roll off. The row marked * is the selected structure; the row marked † is the structure assumed in Section 2.

m	n	k	RMS Log-Error (%)	Max Log-Error (%)	AIC^c^
1	2 †	3	15.09	48.40	−144.63
1	3	4	6.92	35.32	−204.57
1	4	5	6.92	35.32	−201.95
2	3	4	11.03	32.52	−167.21
2	4	5	0.90	2.59	−364.80
2	5	6	0.88	2.40	−364.51
3	4	5	10.51	30.76	−168.43
3	5	6	0.62	1.33	−392.10
3	6	7	0.62	1.30	−389.67
2	4 *	5	0.71	1.71	−384.24

* Second-order-section parameterization of the same pole count, Equation (41).

Two aspects mark the failure as structural. The residuals in Figure 5c are not random but form a single smooth arc, strongly positive at both band edges and uniformly negative in the middle—the signature of asymptotic slopes that are too shallow. And the optimizer drives the two time constants into coincidence, *τ*_1_ = *τ*_2_: a degenerate double pole is the closest a two-pole denominator can come to a roll-off steeper than first order, and a well-known symptom of an under-parameterized denominator. Retaining Equation (33) would anchor the Fisher analysis to a model that misrepresents the response by up to 48%.

### 3.4. Structural Model Selection

The candidate family was widened to

(40) $$H\left(j|\Omega \right)=\frac{K\;{\left(j|\Omega \right)}^{m}}{\prod_{k=1}^{n}\left(1+j\Omega \;\tau_{k}\right)},$$

with m zeros at the origin and n real poles, and every combination with m ∈ {1, 2, 3} and n ∈ {m, …, m + 3} was fitted from 60 randomized starts. Candidate structures are referred to below by the pair (m, n). Model discrimination used the small-sample corrected Akaike information criterion, AIC^c^ [19,20], together with two structural admissibility checks: the residuals must pass a runs test for randomness, and every identified corner frequency must lie inside the measured band, since a corner outside the band is an extrapolation rather than an identified quantity. Both checks were fixed before the scan, a criterion declared in advance. Results are collected in Table 2.

The decisive step in Table 2 occurs between (m, n) = (2, 3) and (2, 4): the root-mean-square error falls by a factor of twelve, from 11.03% to 0.90%, and AIC^c^ drops by almost two hundred units. Adding a fifth pole changes the error by less than 0.03 percentage points, so (2, 4) is the point at which additional structure ceases to buy accuracy. The all-real-pole fit at (2, 4) again returns a coincident pair, *τ*_1_ = *τ*_2_ = 0.6962 s, which signals that the true pole pair lies at the boundary of the real region. Reparameterizing the same four poles as a second-order section plus two real poles,

(41) $$H\left(j|\Omega \right)=\frac{K\;{\left(j|\Omega \right)}^{2}}{\left[1+2\zeta \left(\frac{j\Omega }{\omega_{n}}\right)+{\left(\frac{j\Omega }{\omega_{n}}\right)}^{2}\right]\left(1+j\Omega \tau_{a}\right)\left(1+j\Omega \tau_{b}\right)},$$

removes the boundary constraint at no cost in parameter count and reduces the error further to 0.71% with AIC^c^ = −384.24. Equation (41) is denoted as *M*_2_ and is adopted as the identified dynamic structure.

The structure (3, 5) attains a marginally lower AIC^c^ of −392.10 and would be interpretable as a chain with a second AC-coupling stage, but it is rejected on admissibility grounds: its corner frequencies are 0.0894, 0.1151, 0.2786, 0.4273 and 3.0201 Hz, so one lies below and another sits on the lower band edge, *f_min_* = 0.0999 Hz. Those corners rest on extrapolation, and the accompanying gain, 0.71% to 0.62%, is smaller than the residual scatter. Both structures pass the runs test, so parsimony and in-band support favor M_2_. Extending the sweep below 0.1 Hz would separate them on evidence, as recommended in Section 7. The difference on which the choice partly rests, 0.71% against 0.62%, is smaller than the 0.757% residual scatter, so the ranking is settled by in-band support, not fit quality; the decisive step of Table 2, a twelvefold fall in error, is of a different order. The structures were compared only on the sweep used to fit them.

### 3.5. Identified Dynamic Model and Parameter Uncertainty

**Figure 6:**
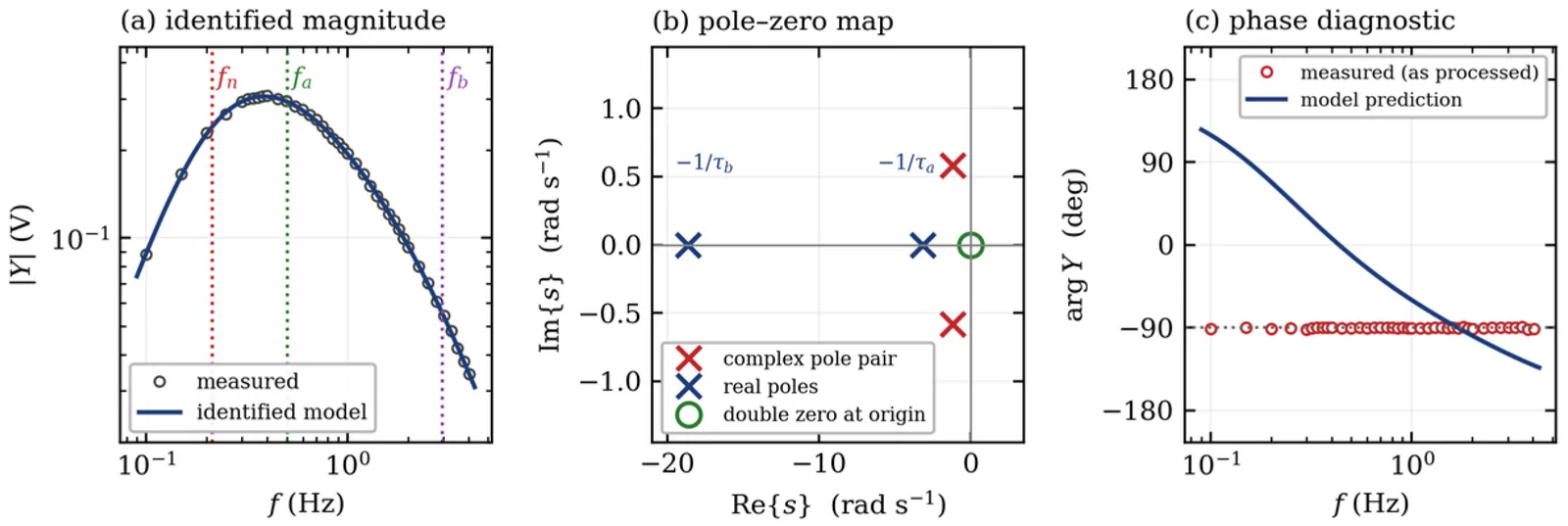
Identified dynamic model *M*_2_. (**a**) Measured magnitude and fitted model, with the three identified corner frequencies marked. (**b**) Pole–zero map: a double zero at the origin, an underdamped complex pole pair at −1.2014 ± 0.5836j rad s^−1^, and two real poles. (**c**) Phase: the model prediction spans more than 200° across the measured band, whereas the phase returned by the present processing is constant at −90.43° ± 0.65°.

**Figure 7:**
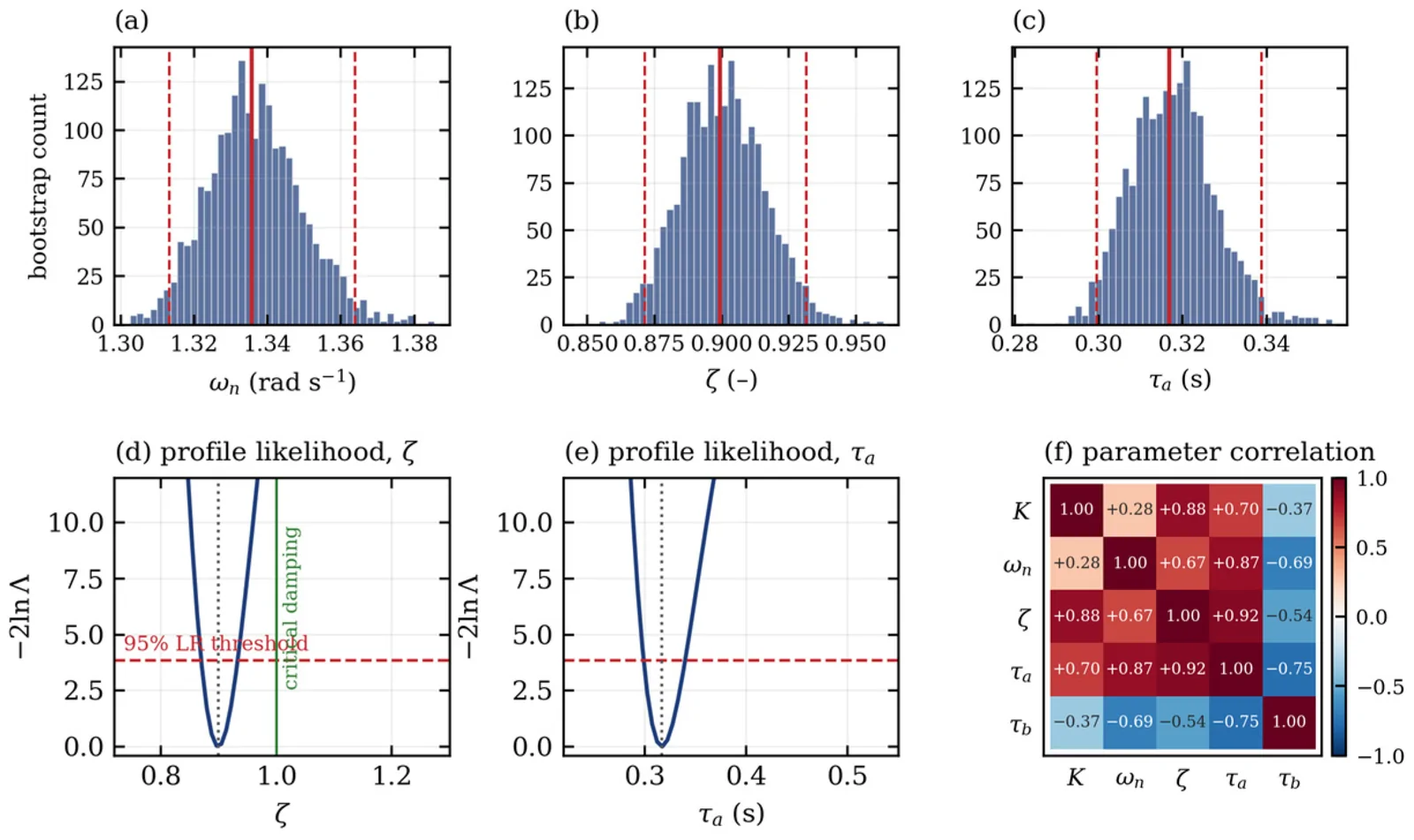
Uncertainty of the identified dynamic parameters. (**a**–**c**) Residual-bootstrap distributions (B = 2000) for *ω_n_*, *ζ* and *τ_a_*; solid and dashed vertical lines mark the point estimate and the 95% interval. (**d**,**e**) Profile likelihood for *ζ* and *τ_a_* with the 95% likelihood-ratio threshold; the interval for *ζ* excludes critical damping. (**f**) Fisher-based parameter correlation matrix for the magnitude-only observation model; the matrix is symmetric by construction, being the normalized inverse of the Gram matrix J_s_^T^J_s_.

The parameters of Equation (41) were estimated by multi-start nonlinear least squares in log-magnitude space. Confidence intervals were obtained by residual bootstrap with B = 2000 resamples [21], and, for the two parameters with the strongest curvature in the objective, independently by profile likelihood with a continuation scheme along the profiled coordinate [14]. Results are given in Table 3, Figure 6 and Figure 7.

The fit reproduces the measured magnitude with a root-mean-square log-magnitude error of 0.71% and a maximum log-magnitude deviation of 1.71% (1.72% in relative terms), corresponding to a residual standard deviation of *σ_r_* = 0.757% of the reading. The residual sequence contains 20 sign runs against an expectation of 20.9 ± 3.1 under the null hypothesis of randomness (z = −0.31) and has a lag-one autocorrelation of 0.073, so no systematic structure remains. The residual magnitude is also comparable at the two ends of the band, 0.80% below 0.5 Hz against 0.63% above 1.5 Hz, which supports the multiplicative error model adopted for the fit.

The bootstrap and profile-likelihood intervals agree closely. Profile likelihood gives *ζ* ∈ [0.8713, 0.9301] and *τ_a_* ∈ [0.3026, 0.3382] s at the 95% level, against bootstrap intervals of [0.87125, 0.93150] and [0.29951, 0.33878] s respectively. The agreement of two methods with different asymptotic assumptions indicates that the estimates are in the practically identifiable regime rather than merely locally identifiable [14,22]. The interval for *ζ* excludes unity by a wide margin, and every one of the 2000 bootstrap replicates returned *ζ* < 1, so the pole pair is genuinely underdamped and the coincident real pair returned by the all-real parameterization is an artifact of that parameterization.

The identified structure has a direct physical reading, summarized in Table 4. A dual-element pyroelectric detector generates a current proportional to the rate of change in absorbed radiation, contributing one zero at the origin and, with the thermal and electrical relaxation of the element, the classical differentiating band-pass characteristic [23,24,25]. An AC-coupled conditioning stage adds a second zero at the origin and a pole, and an output low-pass filter adds the last pole—two zeros and four poles, exactly the structure the data select. Section 2 accounts for the detector alone and omits the conditioning chain, so the discrepancy of Section 3.3 is a specific and correctable modeling omission rather than measurement error. Two structural properties underlie this reading. The model is stable, with all four poles lying in the open left half-plane at −1.2014 ± 0.5836j, −3.1559 and −18.6432 rad s^−1^. And it is minimum-phase, its only zeros being the double zero at the origin; this follows from Equation (41), not the data, since magnitude cannot locate a right-half-plane zero.

Attribution of the two intermediate corners to specific circuit elements is indicative rather than established, since the magnitude data constrain the pole locations but not their physical origin; the count of zeros and poles, however, is determined by the data. The identified transfer function is therefore an effective phenomenological model, not a unique decomposition: structures with the same counts but a different ordering are indistinguishable from any terminal measurement.

### 3.6. Reliability of the Measured Phase

The processing described in Section 3.1 also returns the phase of the first-harmonic coefficient. Across the whole sweep, this phase is −90.43° with a standard deviation of only 0.65°, and the ratio of the real to the imaginary part of the coefficient never exceeds 0.037. Figure 6c contrasts this with the phase predicted by the identified model, which rotates through more than 200° between 0.1 and 4 Hz. A system with four poles and two zeros cannot exhibit a constant phase over such a band, so the measured phase does not describe the sensor.

The cause is the windowing convention. The analysis window of each record was aligned to a zero crossing of the recorded sensor signal itself rather than to the excitation reference. Under that convention, the estimated phase is the phase of the signal relative to its own zero crossing, which is −*π*/2 by construction and independent of the sensor dynamics. The phase information was therefore removed during processing and not by any physical mechanism. Statements about phase below concern what excitation-referenced processing would make available; no measured phase enters any estimate.

The consequence for this work is that the present data set constrains the dynamic parameters through magnitude alone. It also disables one of the arguments advanced in the original design rationale, namely that the excitation band provides both amplitude and phase diversity for separating the dynamic parameters. The remedy is straightforward and does not require new hardware: if the excitation reference is recorded on a second channel, the transfer function can be estimated as the ratio

(42) $$\hat{H}\left(j\Omega_{k}\right)=\frac{Y_{\mathrm{CH}2}\left(k\right)}{Y_{\mathrm{CH}1}\left(k\right)}$$

evaluated on a common window. This restores the phase and, at the same time, divides out any frequency-dependent attenuation of the mechanical aperture actuator, which is otherwise indistinguishable from sensor dynamics at the upper end of the band. Section 4.4 quantifies exactly how much information the phase reference is worth, and Section 7 returns to the actuator question.

### 3.7. Updated Reduced Parameter Vector

With Equation (41) replacing Equation (33), the normalization of Section 2.5 is applied with respect to the double zero,

(43) $${\tilde{Y}}_{i}=\frac{Y_{i}}{K\;{\left(j\Omega_{i}\right)}^{2}},\;\;H_{n}\left(j|\Omega \right)=\frac{1}{\left[1+2\zeta \left(\frac{j\Omega }{\omega_{n}}\right)+{\left(\frac{j\Omega }{\omega_{n}}\right)}^{2}\right]\left(1+j\Omega \tau_{a}\right)\left(1+j\Omega \tau_{b}\right)},$$

and the dynamic block of the parameter vector becomes *θ*_dyn_ = [*ω_n_*, *ζ*, *τ_a_*, *τ*_b_]^T^. Substituting into Equation (37) gives the updated reduced parameter vector

(44) $$\theta ={\left[\alpha_{x}^{*},\;\alpha_{y}^{*},\;\alpha_{z}^{*},\;k_{s},\;c_{1},\;c_{2},\;\omega_{n},\;\zeta ,\;\tau_{a},\;\tau_{b}\right]}^{T}\in R^{10},$$

so that the calibration problem contains six spatial and four dynamic parameters. The dynamic block is now anchored to measured values, *θ*_dyn0_ = [1.33562 rad s^−1^, 0.89949, 0.31687 s, 0.053639 s]^T^, rather than to assumed ones. This is the operating point at which the Fisher analysis of Section 4 is evaluated.

## 4. Fisher-Based Identifiability and Experiment Design

This section evaluates whether the reduced model can be identified from a practical number of aperture-response measurements, and converts that evaluation into an executable calibration plan. The full set of admissible excitation conditions is first treated as a candidate design pool. Local identifiability is assessed on the pool through the scaled Jacobian and the Fisher information matrix, and a greedy D-optimal criterion is then used to select a small subset of informative conditions. The analysis is carried out at the operating point identified in Section 3, so that the conclusions describe the physical module rather than a nominal one.

### 4.1. Scaled Jacobian, Observation Models, and Information Measures

For a design D = {*Ω_i_*}, the complex Jacobian of the model output is

(45) $$J^{c}\left(\theta \right)=\frac{\partial Y\left(\theta \right)}{\partial \theta }\in C^{N\times P}.$$

Because the parameter vector contains quantities with different units and magnitudes—radians per second, seconds, and dimensionless coefficients—a scaled Jacobian is used throughout,

(46) *J_s_* = *J* *D*_0_,  *D*_0_ = diag(*θ*_0_),

so that rank, singular values, and conditioning are assessed in a unit-consistent way, and each column expresses the response to a relative change in the corresponding parameter.

Section 3.5 established that the measurement error on this instrument is multiplicative, with *σ_r_* = 0.757% of the reading. The observation model is therefore written in log-magnitude and phase,

(47) ln|*Y_i_*| = ln|*H*(*jΩ_i_*)| + *e_i_*,  arg*Y_i_* = arg*H*(*jΩ_i_*) + *ε_i_*,

with *e_i_* ∼ N(0, *σ_r_*^2^) and *ε_i_* ∼ N(0, *σ_φ_*^2^). Since a phase estimate obtained from the same complex coefficient inherits the same relative precision, *σ_φ_* = *σ_r_* is taken as the reference. Two observation models are then compared:

Design A (magnitude only), which corresponds to the data actually available, uses the N × P block of derivatives of ln|H|; and Design B (magnitude and phase), which corresponds to the excitation-referenced processing of Equation (42), stacks the log-magnitude and phase blocks to give a 2N × P scaled Jacobian. Design B is a projection, not a measurement: it uses the analytical phase derivatives of the model of Section 3.5, the measured phase having been destroyed as described in Section 3.6; every Design B quantity below is predicted, not observed. The scaled Fisher information matrix follows in both cases as

(48) $$F_{s}=\sigma_{r}^{-2}\;J_{s}^{T}J_{s},$$

and the D-criterion is *Φ^D^* = log det *F_s_*. All information quantities tabulated below include the factor *σ_r_*^−2^ = 1.744 × 10^4^ mV^−2^ of Equation (48), so that *F_s_* carries the units of an inverse variance and *F_s_*^−1^ is directly the Cramér–Rao bound; the singular values *σ_i_*(*J_s_*) reported alongside are those of the unweighted scaled Jacobian and therefore satisfy *λ_i_*(*F_s_*) = *σ_r_*^−2^ *σ_i_*(*J_s_*)^2^. Because *κ* and *η^D^* are ratios, neither is affected by this factor. For comparing designs of different sizes, the D-efficiency of a subset S relative to a reference design D is reported as

(49) $$\eta^{D}\left(S\right)={\left[\frac{\det F_{s}\left(S\right)}{\det F_{s}\left(D\right)}\right]}^{1/\left(2P\right)},$$

which is the ratio of the geometric-mean singular values of the two scaled Jacobians and therefore has a direct interpretation as an average precision ratio [11,26].

### 4.2. Local Identifiability of the Dynamic Block

**Figure 8:**
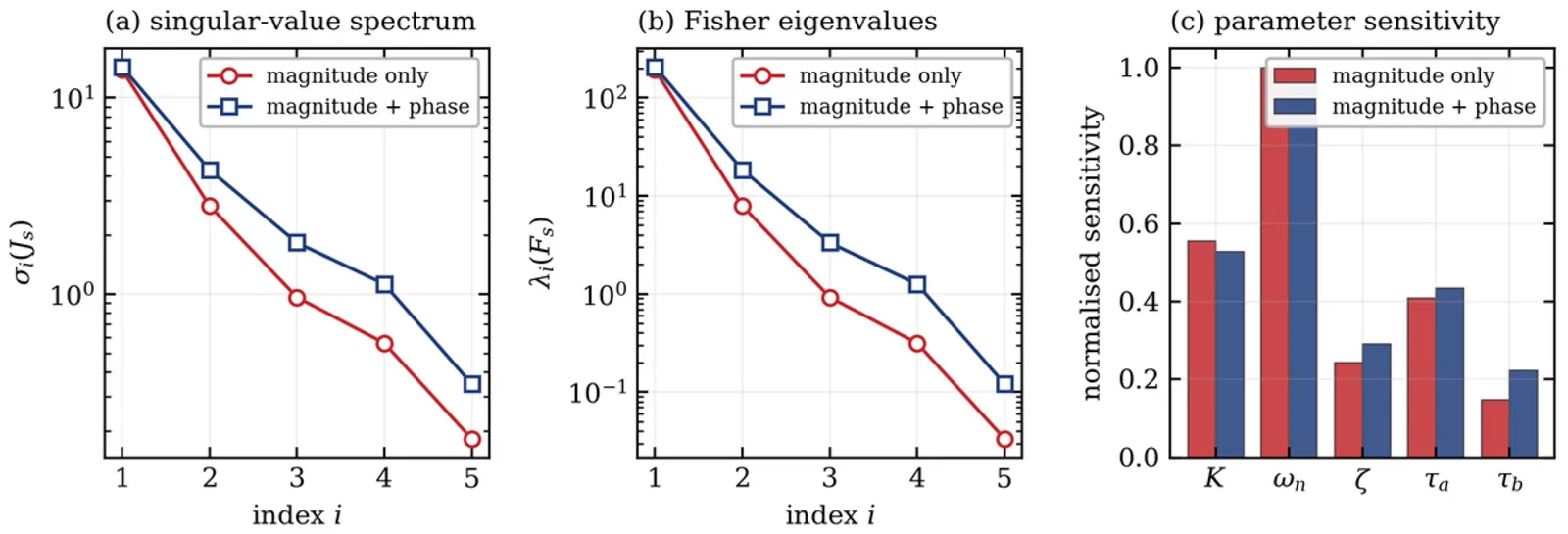
Fisher diagnostics for the dynamic block under the two observation models. (**a**) Singular-value spectra of the scaled Jacobian. (**b**) Eigenvalue spectra of the scaled Fisher matrix. (**c**) Normalized parameter sensitivities.

The frequency sweep of Section 3 constitutes a candidate pool of N = 40 conditions for the dynamic block *θ*_dyn_ augmented by the lumped gain K, so that P = 5. Table 5 reports the identifiability metrics of the full pool under both observation models, and Figure 8 shows the corresponding spectra.

The scaled Jacobian has full column rank under both observation models, so the dynamic block is locally identifiable from the aperture-modulation sweep. More importantly, the condition number is 75.8 for the magnitude-only design and 41.3 with phase. These values are three to four orders of magnitude smaller than the condition numbers typically encountered in the spatial part of this calibration problem, which confirms that the dynamic parameters are the well-determined block of the model and justifies estimating them first and holding them fixed while the spatial parameters are refined. This ordering is exploited in the staged procedure of Section 6.

The singular-value spectrum in Figure 8a spans about two decades without collapsing, consistent with full rank and moderate conditioning. The bootstrap results of Section 3.5 independently check the Fisher analysis: Cramér–Rao bounds under Design A are 1.51, 1.00, 1.85, 3.35 and 1.32% for K, *ω_n_*, *ζ*, *τ_a_* and *τ*_b_, against bootstrap values of 1.40, 0.97, 1.74, 3.19 and 1.20%. Agreement to within about ten per cent on every parameter confirms that the linearized measure predicts the achievable precision—the assumption underlying the whole design argument.

### 4.3. Parameter Sensitivity, Weak Directions, and Correlation

Normalized column norms of *J_s_* rank the parameters by their influence on the predicted response, and the right singular vector associated with *σ_min_*(*J_s_*) identifies the least observable combination. Both are reported in Table 6 together with the Cramér–Rao bounds and the bootstrap standard deviations.

The response is most sensitive to *ω_n_*, which fixes the band-pass peak and is therefore the best-determined parameter, and least sensitive to *τ*_b_, whose corner at 2.97 Hz lies near the upper band edge where the amplitude has fallen to about a ninth of its peak. The weakest identifiable direction is dominated by *τ_a_* (−0.804) and *ζ* (−0.425). This is physically meaningful: the real pole at 0.50 Hz and the complex pair at 0.21 Hz act on the same side of the maximum, so widening the pair can be partly offset by shifting the real pole. Correspondingly, *ρ*(*ζ*, *τ_a_*) = 0.918 is the largest entry in Figure 7f, followed by *ρ*(*ω_n_*, *τ_a_*) = 0.872 and *ρ*(K, *ζ*) = 0.881. The two poles straddling the peak are the coupled pair of this problem, as translation and tilt are spatial ones.

Adding phase reduces the coupling without removing it, and every parameter benefits: *ρ*(*ζ*, *τ_a_*) falls to 0.891, *ρ*(K, *ζ*) to 0.804, and the bound on *τ_a_* improves from 3.35% to 1.82%. The reason is geometric—magnitude and phase respond to a pole location along orthogonal directions in the complex plane, so a phase measurement supplies exactly the information a magnitude measurement discards.

### 4.4. Projected Information Value of the Excitation Phase Reference

The comparison of Designs A and B in Table 5 quantifies what the processing correction of Equation (42) is predicted to be worth; as Section 4.1 states, this is a projection, not an empirical comparison. Recovering the phase raises log det *F_s_* from 51.5365 to 56.4024, a factor of 130 in the determinant, equivalent to a factor of 2.65 in the geometric-mean singular value. It also lowers the condition number of the scaled Jacobian from 75.8 to 41.3 and raises *λ_min_*(*F_s_*) by a factor of 3.6, so the improvement is not concentrated in the already well-determined directions but falls mainly on the weak ones.

Expressed in experimental terms, the effect is substantial. As shown in Section 4.5, a D-optimal complex design of twelve frequencies attains log det *F_s_* = 51.812, which already exceeds the value of 51.537 achieved by all forty magnitude-only measurements. Correcting the windowing reference therefore reduces the number of frequency settings required for a given information content by roughly a factor of three, at no cost in hardware and with no additional measurement time. Among all the design changes examined in this work, this is the one with the largest projected ratio of benefit to effort, conditional on the assumptions of Section 4.1.

### 4.5. Greedy D-Optimal Subset Selection

Performing every candidate condition is impractical once the aperture and pose dimensions are included, so a reduced subset is selected using the D-optimal criterion. For a selected subset S, the scaled Fisher matrix is *F_s_*(S) = *J_s_*(S)^T^ *J_s_*(S), and the criterion to be maximized is

(50) *Φ^D^*(*S*) = logdet[*F_s_*(*S*) + *εI*],

where *ε*I is a small regularization term used for numerical stability. Exact combinatorial optimization is intractable, so a greedy sequential selection is used: at iteration k + 1, the next condition is

(51) $$i_{k+1}=\mathrm{argmax}\left\{\mathrm{logdet}\left[F_{s}\left(S_{k}\right)+\Delta F_{i}+\mathrm{\varepsilon I}\right]:i\in D\backslash S_{k}\right\},\;\;\Delta F_{i}=J_{s,i}^{T}J_{s,i}.$$

**Figure 9:**
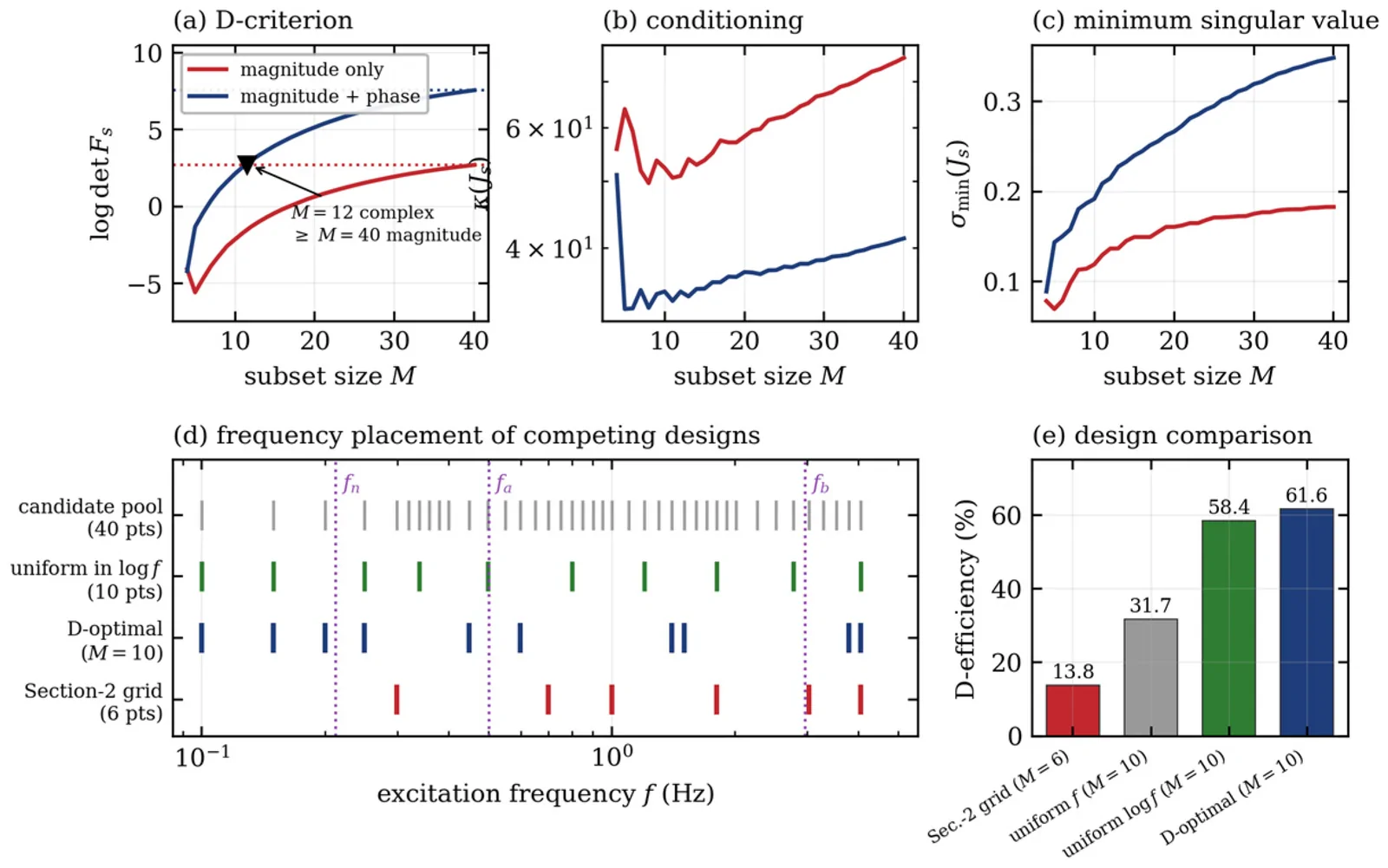
D-optimal experimental design on the measured candidate pool. (**a**) D-criterion against subset size for the two observation models; the marker indicates the size at which a complex design overtakes the full 40-point magnitude-only design. (**b**) Condition number and (**c**) minimum singular value of the scaled Jacobian against subset size. (**d**) Frequency placement of the competing designs relative to the three identified corner frequencies. (**e**) D-efficiency of the designs of Table 9.

Because *Φ^D^* is a monotone submodular set function, the greedy sequence is guaranteed to attain at least a fraction 1 − 1/e of the optimum of the corresponding coverage problem, which is why greedy selection is the standard practical choice for this criterion [27]. To prevent the selected subset from concentrating in a single region of the band, an initial coverage step is applied in which one condition is drawn from each of four frequency bands spanning the pool, after which the remaining conditions are added greedily. Results are reported in Table 7 and Figure 9.

Every subset down to M = 5 retains full column rank, so local identifiability of the dynamic block is not the binding constraint; the binding constraint is precision. D-efficiency rises steeply up to about M = 15 and then flattens, so a subset of ten to fifteen frequencies captures the majority of the information available in the full pool while reducing the measurement effort by 62% to 75%. The D-optimal selection at M = 10 is

(52) *f* ∈ {0.100, 0.150, 0.200, 0.250, 0.450, 0.600, 1.404, 1.505, 3.790, 4.045} Hz,

which places four points on the low-frequency rising flank, two near the response maximum, and two pairs near the upper end of the band. The pattern is the one expected from the sensitivity analysis: points are clustered where the model derivatives differ most strongly from one another, that is, on the flanks rather than at the peak, and the two clusters at the ends of the band are what allow the coupled pair *ζ* and *τ_a_* to be separated.

The corresponding results for the complex observation model are given in Table 8. The condition number is markedly lower at every subset size, and it is essentially flat in M, which indicates that with phase available, the design problem is no longer dominated by the need to break parameter couplings.

**Table 8:** Greedy D-optimal subsets under the complex observation model of Equation (42). D-efficiency is referred to the full 40-point complex design.

M	σ_min_(J_s_)	κ(J_s_)	λ_min_(F_s_) (mV^−2^)	Log Det F_s_	η^D^ (%)
5	1.438 × 10^−1^	32.52	3.606 × 10^2^	47.509	41.1
8	1.805 × 10^−1^	32.65	5.681 × 10^2^	49.913	52.3
10	1.918 × 10^−1^	34.50	6.417 × 10^2^	50.963	58.0
12	2.147 × 10^−1^	34.49	8.042 × 10^2^	51.812	63.2
15	2.399 × 10^−1^	34.82	1.004 × 10^3^	52.789	69.7
20	2.665 × 10^−1^	36.83	1.239 × 10^3^	53.990	78.6
40	3.484 × 10^−1^	41.25	2.117 × 10^3^	56.402	100.0

**Table 9:** Comparison of candidate excitation designs under the magnitude-only observation model, evaluated at the identified operating point.

Design	M	κ(J_s_)	Log Det F_s_	η^D^ (%)
Nominal grid of Section 2, 0.3–5 Hz	6	3550.05	31.764	13.8
Greedy D-optimal	6	59.16	44.131	47.7
Uniform in f	10	538.95	40.056	31.7
Uniform in log f	10	61.83	46.164	58.4
Greedy D-optimal	10	52.32	46.688	61.6
Full candidate pool	40	75.82	51.537	100.0

### 4.6. Comparison with Heuristic Designs

The practical value of the D-optimal construction is best judged against the designs an experimenter would otherwise adopt. Table 9 compares four alternatives evaluated on the same information measure.

The nominal grid of Section 2, spanning 0.3–5 Hz, achieves only 13.8% D-efficiency with *κ* = 3.55 × 10^3^; a greedy D-optimal design of the same size is 3.5 times more efficient and 60 times better conditioned. Figure 9d shows why: the identified pole pair sits at *f_n_* = 0.213 Hz, below the lower limit of the grid, so the rising flank is never sampled and the pair must be inferred by extrapolation, while the uppermost point at 5 Hz lies where the response has fallen to a tenth of its peak. The grid was chosen for a system whose corners were assumed near 1.1 and 5.3 Hz; the measured corners are 0.21, 0.50 and 2.97 Hz.

A geometric grid is a good heuristic: uniform spacing in log f reaches 58.4% against 61.6% for D-optimal selection of the same size, so formal optimization adds little once the band is right. Uniform spacing in f reaches only 31.7% and is an order of magnitude worse conditioned, wasting most points on the shallow upper tail. Choosing the correct band therefore matters far more than placement within it—and the band can only be chosen once the structure is known, which is the argument for performing Section 3 before Section 4.

### 4.7. Extension to the Full Spatial–Dynamic Design Pool

The analysis above treats the dynamic block in isolation because the frequency sweep of Section 3 varies only the excitation frequency. The full calibration problem of Equation (44) additionally requires excitation of the six spatial parameters, which is obtained by varying the aperture operating radius *a*_0_ and the pose p. The candidate pool is then the Cartesian product

(53) *D* = {*a*_0_} × {*A_m_* = 0.1 *a*_0_} × {*f*} × {*p*},

and the complex Jacobian *J^c^* ∈ ℂ^{N × 10} is assembled by differentiating Equation (36) with respect to all ten parameters, with the real and imaginary parts stacked as in Section 4.1. The selection procedure of Equations (50) and (51) applies unchanged, with the coverage step extended so that one condition is drawn from each (*a*_0_, f) pair before greedy selection proceeds.

Three properties of the full problem follow. First, the dynamic and spatial blocks enter Equation (36) multiplicatively, so a design informative for one is not automatically informative for the other; the frequency axis must be chosen for the dynamic block and the aperture and pose axes for the spatial block, which the coverage step enforces. Second, the dynamic block is comparatively well conditioned, *κ* ≈ 76 against values orders of magnitude larger for the spatial block, motivating the staged strategy of Section 6. Third, the frequency axis should be taken from Equation (52) rather than the nominal grid, since the informative band does not depend on aperture or pose.

Completing the full-pool analysis requires the spatial Jacobian block ∂(∂g/∂a)/∂*θ*, which depends on module-specific constants obtained from manufacturer data and the mechanical setup, so the combined-pool metrics must be recomputed per device at the operating point of Section 3. Section 5 approaches the same block from the opposite direction, identifying the reference pose experimentally and testing whether the couplings predicted here appear in measured data.

## 5. Experimental Identification of the Spatial Response

Section 4.7 deferred the spatial block because the sweep of Section 3 holds the aperture radius and pose fixed and so carries no information about them. This section closes that gap: on the same bench the six alignment parameters are varied directly while the aperture is modulated at a fixed frequency, and the response is analyzed by a two-stage design of experiments. The purpose is to locate the reference pose p* experimentally, and to test whether the couplings predicted by the Fisher analysis of Section 4.3 appear in measured data.

### 5.1. Response Variable and Experimental Strategy

The differential kernel of Equation (15) is antisymmetric about the optical axis. At perfect alignment, the aperture-integrated response therefore vanishes, and the calibration problem becomes a null-seeking one: the reference pose is the pose at which the modulated response is smallest. This inverts the usual sense of an optimization criterion and makes the objective a minimum rather than a maximum.

The response is quantified by the power spectral density of the output voltage evaluated at the modulation frequency [28], and the regression variable is its square root, which linearizes the relation between radiant power and voltage and gives a more nearly constant variance across the design space. The six factors are the three translations *Δ*x, *Δ*y, *Δ*z of the microstage and the three rotations *θ*_x_, *θ*_y_, *θ*_z_ introduced in Section 2.1.

A full quadratic model in six factors has 28 coefficients, so one design over a wide domain would be both large and badly placed. A two-stage strategy is used instead: a definitive screening design [29] explores a coarse domain, identifies the active factors and relocates the origin; a D-optimal design [26,30,31] then builds an accurate surface in a narrow domain around it. Terms are selected at each stage by forward selection on the corrected Akaike information criterion, the same criterion used for the dynamic structure in Section 3.4.

### 5.2. Screening Stage

The screening design comprises 17 runs: the twelve fold-over pairs required by a definitive screening design with six factors, together with five replicated center points. The design and the measured response are given in Table 10.

The five center replicates give a model-free estimate of repeatability: on the square-root scale their standard deviation is 6.11 mV against a mean of 26.68 mV, so the complete measure-and-reposition cycle repeats to about 23%. This sets the resolution against which every subsequent improvement must be judged, and no comparable estimate is available from the sweep of Section 3. The two dispersion figures are not interchangeable: this one covers a full measure-and-reposition cycle; the 0.757% of Section 3.5 is a fit residual at a fixed pose. It is this one that belongs in a joint uncertainty budget.

**Figure 10:**
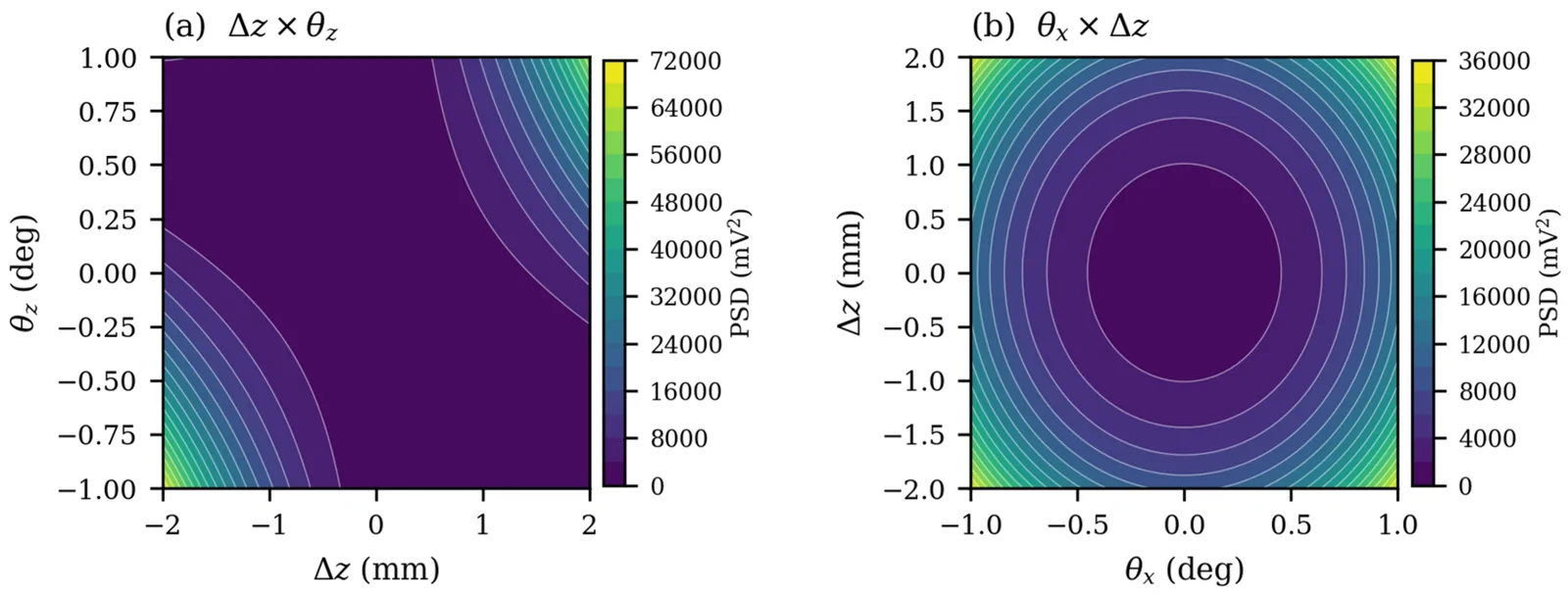
Response surfaces predicted by the fitted screening model of Table 11, evaluated on the power-spectral-density scale with all remaining factors held at the design center. The surfaces are model predictions, not interpolated measurements; the 17 underlying measured runs are listed in Table 10. (**a**) The *Δ*z × *θ*_z_ interaction produces a twisted valley: axial displacement can be traded against roll over a wide range at almost constant response. (**b**) The *θ*_x_ × *Δ*z section shows the quadratic bowl formed by the *θ*_x_^2^ and *Δz*^2^ terms, which fixes an interior optimum.

Forward selection retains six terms. The fitted model attains *R*^2^ = 0.9942 and adjusted *R*^2^ = 0.9907, with F(6, 10) = 283.8 and *p* = 1.4 × 10^−10^; the residual standard error is 11.24 mV, about 1.8 times the pure error, indicating mild lack of fit but no gross structural failure. Coefficients are listed in the upper block of Table 11, and the fitted surface is shown in Figure 10.

**Table 11:** Coded regression coefficients retained by forward selection at the two stages. Coefficients refer to the square root of the power spectral density with factors coded to the interval [−1, 1].

Stage	Term	Coefficient (mV)	t-Ratio	*p*-Value
Screening	intercept	26.48	5.39	0.0003
Screening	Δz × θ_z_	154.87	30.82	<0.0001
Screening	θ_x_^2^	88.47	12.52	<0.0001
Screening	Δz^2^	71.31	10.09	<0.0001
Screening	Δx	25.82	7.27	<0.0001
Screening	Δx × Δy	22.53	4.50	0.0012
Screening	θ_z_	−8.98	−2.53	0.0300
Optimization	intercept	36.10	5.83	<0.0001
Optimization	θ_x_ × θ_y_	−36.18	−13.46	<0.0001
Optimization	θ_y_ × θ_z_	13.77	5.25	<0.0001
Optimization	θ_y_	12.49	5.28	<0.0001
Optimization	θ_y_^2^	26.76	5.18	<0.0001
Optimization	θ_x_	−13.86	−5.58	<0.0001
Optimization	Δx × θ_y_	7.38	2.92	0.0096
Optimization	θ_x_ × θ_z_	−8.97	−3.27	0.0045
Optimization	Δx × θ_x_	−6.74	−2.33	0.0323
Optimization	θ_x_^2^	14.35	2.89	0.0101
Optimization	Δz^2^	−14.57	−2.84	0.0114

Two features of Figure 10 govern the outcome. The *Δ*z × *θ*_z_ interaction is by far the strongest single effect (t = 30.8) and produces the twisted valley of Figure 10a: over the coarse domain the axial position and the roll angle compensate each other almost completely, so neither is separately determined. The pure quadratic terms *θ*_x_^2^ and *Δz*^2^, on the other hand, are both positive and large, and they close the valley in the orthogonal directions, giving the bowl of Figure 10b and hence a well-defined interior minimum. Relocating to that minimum reduces the response from 558.45 to 300.28 mV^2^.

### 5.3. Optimization Stage

The screening optimum is adopted as the new origin, and a 28-run D-optimal design is generated over a domain narrowed to ±0.1 mm in translation and ±0.5° in *θ*_x_ and *θ*_z_, with ±0.1° in *θ*_y_. The design and the measured response are given in Table 12.

**Figure 11:**
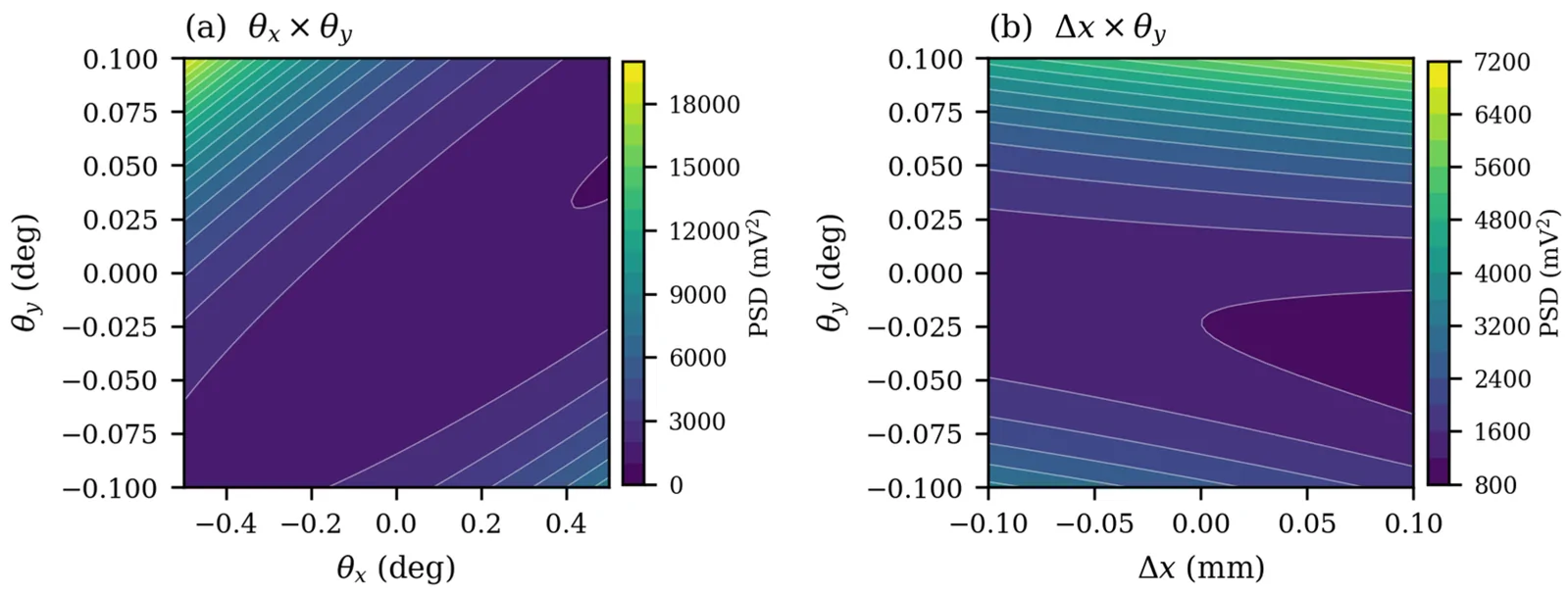
Response surfaces predicted by the fitted optimization-stage model of Table 11, with all remaining factors held at the design center; the 28 underlying measured runs are listed in Table 12. (**a**) The *θ*_x_ × *θ*_y_ interaction dominates the narrow domain and forms a narrow diagonal valley rather than an isolated point minimum. (**b**) The *Δ*x × *θ*_y_ section is the measured signature of translation–tilt coupling: a lateral offset can be compensated by a tilt about the orthogonal axis.

Ten terms are retained, giving *R*^2^ = 0.9593 and adjusted *R*^2^ = 0.9354, with F(10, 17) = 40.1 and *p* = 7.8 × 10^−10^ and a residual standard error of 10.79 mV. The character of the model changes completely between the two stages. Over the coarse domain, the response was dominated by axial translation; in the narrow domain, the rotations take over, with *θ*_x_ × *θ*_y_ the largest term (t = −13.5), followed by *θ*_y_^2^, *θ*_y_ and *θ*_x_. The fitted surface is shown in Figure 11. The translations survive only through their interactions with the rotations, a change in regime that is characteristic of response surfaces explored at two very different scales [31].

Figure 11b connects this section to Section 4. The *Δ*x × *θ*_y_ interaction is significant (t = 2.92, *p* = 0.010), as is *Δ*x × *θ*_x_ (t = −2.33, *p* = 0.032): a lateral displacement of the element can be partly compensated by a tilt about the orthogonal axis, so the two are not separately resolved by the aperture-integrated response. This is precisely the translation–tilt coupling identified as a weak direction of the scaled Jacobian in Section 4.3, observed here directly in measured data rather than inferred from a linearization. Geometrically, it is the Abbe offset: rotating about an axis that misses the sensing element converts an angular error into a transverse one. 

### 5.4. Verification at the Identified Null

**Figure 12:**
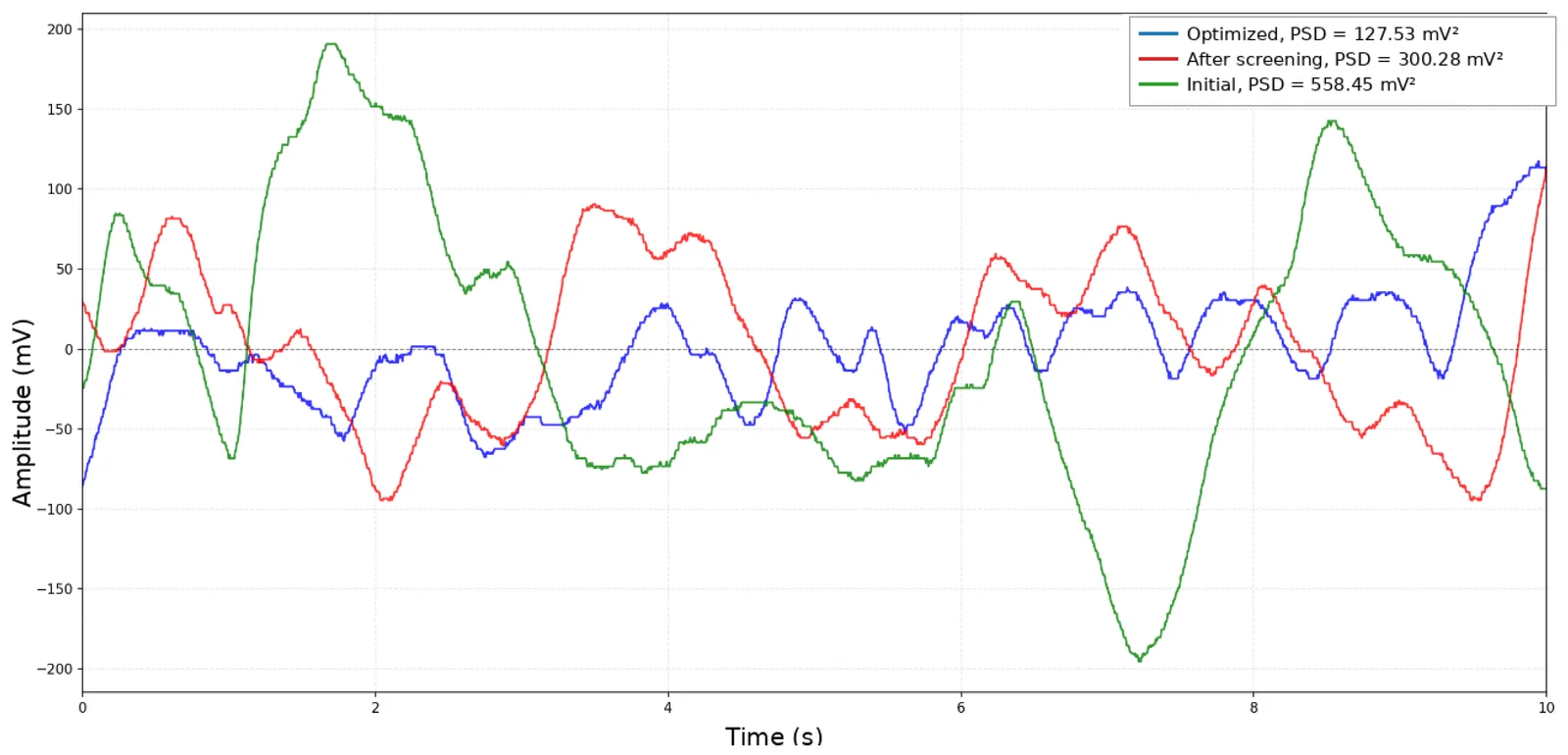
Output voltage over 10 s at the three alignment states, with the aperture modulated at 1 Hz. The peak-to-peak envelope contracts from about ±200 mV at the initial alignment to about ±35 mV at the identified null, and the residual waveform loses its periodic character.

The alignment coordinates and the measured response at each stage are collected in Table 13, and the corresponding time-domain records are compared in Figure 12.

The overall reduction is a factor of 4.4 in power spectral density, from 558.45 to 127.53 mV^2^. Expressed on the regression scale, this is a change from 23.63 to 11.29 mV, which is 2.0 times the pure-error standard deviation of 6.11 mV established in Section 5.2. The improvement is therefore resolved, but not by a large margin, and each individual stage advances the null by only about one standard deviation. Replication at the identified null would strengthen the claim, and is recommended.

Figure 12 shows that the reduction is qualitative as well as quantitative. At the initial alignment, the record is dominated by a clean 1 Hz oscillation, the signature of a residual differential response to the modulated aperture. At the identified null, the periodic component is no longer visually distinguishable, and the remaining trace is irregular, which is what the model predicts when the antisymmetric kernel is centered on the aperture axis and the surviving signal is drift and electronic noise rather than modulated radiation.

### 5.5. Consequences for the Reduced Model

Three conclusions follow. The reference pose is locatable in practice: 45 runs locate a null that is stable and reproducible to within the measurement repeatability. Locatable is not identifiable component by component: the null does not separate the components along the translation–tilt direction. The response surface near that null is quadratic and, in the narrow domain, well conditioned, supporting the local linearization on which Section 4 rests. And the translation–tilt coupling predicted by the scaled Jacobian is confirmed experimentally, so translation and rotation are not separately resolved by a scalar response.

That last point delimits what this experiment can deliver. The power spectral density is a scalar summary that discards the sign structure of the kernel and retains no phase, so it locates the null but cannot separate the parameters that trade off along it. Full identification of Equation (44) requires the complex model of Equation (36) over a design varying aperture radius as well as pose, estimated by the procedure of Section 6.

## 6. Structured Estimation Procedure

The identifiability analysis shows that the reduced model contains parameter blocks of very different conditioning. The estimation procedure is organized to exploit that structure rather than to ignore it. It is reported as a proposal: no joint spatial–dynamic estimation has yet been run on measured data.

### 6.1. Separable Structure of the Residual Coefficients

Equation (28) established that the residual part of the aperture derivative is linear in the coefficients c = [*c*_1_, *c*_2_]^T^ for fixed values of the remaining parameters. Collecting the nonlinear block as

(54) $$\eta ={\left[\alpha_{x}^{*},\;\alpha_{y}^{*},\;\alpha_{z}^{*},\;k_{s},\;\omega_{n},\;\zeta ,\;\tau_{a},\;\tau_{b}\right]}^{T},$$

the model output for the i-th condition can be written as

(55) $${\tilde{Y}}_{i}\left(\eta ,\;c\right)=u_{i}\left(\eta \right)+v_{i}{\left(\eta \right)}^{T}c,$$

where *u_i_*(*η*) collects the physics-based contribution *A_m_*,_i_ *H_n_*(jΩ_i_) ∂g_phys_/∂a and *v_i_*(*η*) collects the residual basis integrals *A_m_*,_i_ *H_n_*(jΩ_i_) *b_m_*(*a*_0_,_i_, *p_i_*; *η*) of Equation (29). Stacking over all conditions and separating real and imaginary parts gives the residual vector

(56) *r*(*η*, *c*) = *z* − *u*(*η*) − *V*(*η*) *c*,

where z is the vector of measurements. This formulation constitutes a separable nonlinear least-squares problem that can be addressed using variable-projection methods [15,16].

### 6.2. Regularized Variable-Projection Subproblem

The residual coefficients are the weakly determined block of the model: they act as a correction to a kernel that already reproduces the dominant bipolar structure, and their basis functions are by construction small in the region where the physics-based kernel is large. Leaving them unconstrained would allow the correction to absorb variation that properly belongs to the pose and envelope parameters. They are therefore updated through a regularized linear subproblem,

(57) $$\hat{c}\left(\eta \right)=\arg\min_{c}\left\{{∥z-u\left(\eta \right)-V\left(\eta \right)c∥}^{2}+\lambda {∥c∥}^{2}\right\}={\left[V^{T}V+\mathrm{\lambda I}\right]}^{-1}V^{T}\left[z-u\left(\eta \right)\right],$$

whose solution is available in closed form. Substituting $\hat{c}$(*η*) back into Equation (56) yields the reduced objective

(58) $$\Phi \left(\eta \right)={∥z-u\left(\eta \right)-V\left(\eta \right)\;\hat{c}\left(\eta \right)∥}^{2},$$

which depends on the nonlinear block alone. The dimension of the nonlinear search is thereby reduced from ten to eight, and the linear block is guaranteed to be at its conditional optimum at every iteration. The regularization weight *λ* is selected by generalized cross-validation on the reduced objective; because *λ* acts only on the weak block, the sensitivity of the remaining estimates to its value is small.

### 6.3. Staged Estimation Algorithm

The complete procedure exploits the block structure established in Section 4 and proceeds in four stages.

Stage 1. Dynamic initialization: The dynamic parameters *ω_n_*, *ζ*, *τ_a_*, *τ*_b_ and the lumped gain are estimated from a single-aperture frequency sweep at the D-optimal frequencies of Equation (52), using the multi-start log-magnitude procedure of Section 3.5. Because the aperture and pose are held fixed, the spatial factor is constant and does not need to be known.
Stage 2. Spatial estimation with frozen dynamics: Holding *θ*_dyn_ at the Stage-1 estimate, the pose and envelope parameters α_x_*, *α*_y_*, *α*_z_*, *k_s_* are estimated from the D-optimal aperture–pose–frequency subset, with the residual coefficients eliminated at every iteration by Equation (57). Freezing the well-conditioned block prevents the poorly conditioned spatial directions from corrupting it.
Stage 3. Joint refinement: All eight nonlinear parameters are released, and Equation (58) is minimized by a trust-region Gauss–Newton method started from the Stage-2 estimate. The scaled Jacobian is monitored at each iteration, and the step is restricted along directions whose singular value falls below a preset fraction of *σ_max_*.
Stage 4. Uncertainty quantification: Confidence intervals are obtained by residual bootstrap as in Section 3.5, and profile likelihood is computed for any parameter whose bootstrap interval is asymmetric or whose Fisher-based bound disagrees with the bootstrap standard deviation by more than a factor of about 1.5.

The staging is not a numerical convenience but a direct consequence of the identifiability structure. Section 4.2 established that the dynamic block is three to four orders of magnitude better conditioned than the spatial block, and Section 3.3 showed that a single-start optimizer applied to the dynamic block alone can converge to a degenerate solution. Attempting a single joint optimization from an arbitrary start therefore risks both failure modes at once, which is the standard argument for staged identification of structured models [18,32]. Stage 1 accordingly uses randomized starts; Stages 2 and 3 start from the preceding stage, and convergence is declared when the relative change in the reduced objective falls below 10^−8^ and the scaled gradient norm below 10^−6^.

## 7. Discussion

### 7.1. Why the Assumed Dynamic Structure Failed

Equation (33) is the textbook description of a pyroelectric detector, and correct as such [23,24]. What it omits is the conditioning chain inseparable from a commercial module. The identified structure differs by exactly one AC-coupled stage: one extra zero at the origin and one extra pole. The discrepancy is therefore not a failure of the physical reasoning in Section 2 but a consequence of drawing the system boundary around the detector rather than the module—and since aperture calibration accesses the module output, not the element current, the boundary must enclose the electronics.

This generalizes to packaged sensors: the asymptotic slopes of a measured frequency response are a structural fingerprint readable before any parameter is estimated, and invariant to the unknown spatial factor provided aperture and pose are held constant. Checking them costs one sweep and can invalidate a structure that would otherwise propagate silently through an entire identifiability analysis.

### 7.2. Implications for Calibration Design

Table 9 shows that the dominant design decision is the excitation band, not the placement within it. Moving from 0.3 to 5 Hz to a band bracketing the identified corners raises D-efficiency from 13.8% to 58.4% using only geometric spacing; formal D-optimal selection adds three points more. Since the band cannot be chosen without the corner frequencies, and those require an adequate structure, the ordering used here—structure, then information, then design—is necessary rather than merely convenient.

The second design conclusion concerns the phase reference. Section 4.4 showed that excitation-referenced processing multiplies the determinant of the Fisher matrix by 130 and reduces the number of frequency settings needed for a given information content by roughly a factor of three. This is a processing change, not a hardware change, and it should be regarded as a prerequisite rather than an improvement.

### 7.3. Limitations

Five limitations of the present data set should be stated explicitly.

First, the sweep was performed at a single-aperture operating radius and a single pose, so it constrains the dynamic block only. The spatial parameters of Equation (44) remain unvalidated against measurement, and the full-pool metrics of Section 4.7 are therefore reported as a procedure rather than as results.
Second, the phase information was destroyed by the windowing convention, as analyzed in Section 3.6. All dynamic estimates reported here rest on magnitude alone. The agreement between bootstrap and profile-likelihood intervals indicates that this is sufficient for practical identifiability of the four dynamic parameters, but it is a weaker basis than a complex measurement, and it leaves the coupling *ρ*(*ζ*, *τ_a_*) = 0.918 larger than it needs to be.
Third, the time-domain records show visible harmonic distortion at low excitation frequencies, with a second-harmonic component of order five to fifteen per cent of the fundamental below 0.4 Hz and a corresponding waveform asymmetry, whereas Equation (31) presumes it is negligible. The distortion may originate in the aperture kinematics or in a genuine nonlinearity of the aperture-response function at the modulation depth used, and the two cannot be separated from the sensing channel alone. The modulation depth used throughout is *A_m_*/*a*_0_ = 0.1, small enough for the first-order expansion in principle; the total harmonic distortion should nevertheless be measured directly and the depth reduced if it exceeds a few per cent. A second harmonic is orthogonal to the projection of Section 3.1 and does not bias the estimated amplitude. It does indicate that Equation (31) is incomplete: curvature of the aperture response feeds back at second order in the modulation depth, about one per cent here. The residual does not rise there, 0.80% against 0.63% above 1.5 Hz, so no such error is evident.
Fourth, the amplitude of the mechanical aperture modulation was not measured independently. If the actuator fails to reach its commanded amplitude at the upper end of the sweep, part of the identified roll-off is mechanical rather than sensory, and *τ*_b_ in particular would be biased. Excitation-referenced processing removes this ambiguity as a by-product, since the actuator response then appears in both the numerator and denominator of Equation (42). Refitting Equation (41) to a synthetic response carrying a first-order mechanical attenuation bounds it: a corner at 10 Hz displaces the highest identified corner from 2.967 to 2.613 Hz (11.9%), leaves the natural frequency and damping ratio within 2%, and gives a refit residual of 0.305%, below the 0.757% scatter, so such a bias would be invisible in the fit. That corner is therefore a lower bound on the sensor’s own.
Fifth, the noise level underlying the Fisher analysis is not an independent measurement. The 0.757% adopted in Section 4.1 is the fit residual of Section 3.5, confounded with model discrepancy and bench drift, so the Cramér–Rao bounds of Table 6 are optimistic to that degree. The residual bootstrap inherits the same assumption; profile likelihood does not, and agrees with both.

### 7.4. Extensions

Five extensions follow. Extending the sweep below 0.1 Hz would bring the lowest corner well inside the band and let M_2_ and (3, 5) be discriminated on evidence rather than admissibility. Replicated measurements at a few frequencies would estimate the measurement noise directly, which is presently inferred from the fit residual and thus confounded with model error. And since F_s_ is evaluated at a single operating point, a robust design averaging the criterion over the confidence region of Table 3 would guard against the sensitivity identified in Section 4.6. One sweep with the excitation reference on a second channel would restore the phase, test the projection of Section 4.4 and remove the actuator ambiguity of Section 7.3; measuring the conditioning circuit would make the attribution of Table 4 an identified decomposition.

## 8. Conclusions

This paper has developed a physics-informed framework for identifying the spatial and dynamic responses of dual-element PIR sensors from variable circular-aperture excitation, and has carried the dynamic half of that framework through to experimental identification and experiment design. The spatial half rests only on the reference-pose location of Section 5; the joint estimation of Section 6 is specified but not demonstrated.

The dynamic structure assumed on physical grounds—one zero at the origin and two poles—proved structurally inadequate: fitted to a 40-point sweep, it leaves a root-mean-square log-magnitude error of 15.1% with a maximum deviation of 48.4% (62% in relative amplitude), and systematic rather than random residuals. A structural scan selected a model with two zeros at the origin and four poles, arranged as an underdamped complex pair and two real poles, reproducing the measurement to 0.71% root-mean-square and 1.71% maximum. The identified corners are 0.2126, 0.5023 and 2.9672 Hz with damping ratio 0.899, the extra zero and pole being attributable to the AC-coupled conditioning stage.

Evaluated at this identified operating point, the scaled Jacobian of the dynamic block has full column rank with a condition number of 75.8 from magnitude data alone, and the Cramér–Rao bounds agree with residual-bootstrap standard deviations to within about ten per cent on every parameter. The weakest identifiable direction couples the damping ratio to the first real pole, with a correlation of 0.918, reflecting that both act on the same side of the response maximum.

Ten greedily selected D-optimal frequencies retain 61.6% of the D-efficiency of the full 40-point sweep, whereas the grid originally proposed for this instrument achieves 13.8% with a condition number sixty times larger, because it does not sample the band containing the identified pole pair. Restoring the excitation phase reference—a change in processing, not hardware—multiplies the Fisher determinant by 130.

Taken together, these results argue for a specific ordering of the calibration workflow: identify the model structure from measurement, evaluate the information content at the identified operating point, and only then select the experiments. Applied in this order, the framework converts a candidate design pool into a small, executable calibration plan while making the weakly observable directions of the problem explicit rather than implicit.

## Figures and Tables

**Table 1:** Configuration of the dynamic-response frequency sweep.

Quantity	Value
Number of excitation frequencies	40
Frequency range	0.0999–4.0453 Hz
Sampling rate f_s_	500.09 ± 0.06 Hz
Analysis window	15 excitation cycles (coherent)
Relative frequency resolution Δf/f	1/15 = 6.7%
Aperture operating radius a_0_	fixed (single setting)
Modulation amplitude A_m_	fixed, A_m_/a_0_ = 0.1
Pose p	nominal, not varied
Observed peak response	0.3068 V at 0.3996 Hz
Observed dynamic range	8.95:1

**Table 3:** Identified dynamic parameters of *M*_2_ with 95% confidence intervals from residual bootstrap (B = 2000). Derived corner frequencies are reported below the rule.

Parameter	Estimate	95% CI	Relative Std. (%)
K (V s^2^)	0.26352	[0.25698, 0.27104]	1.40
ω_n_ (rad s^−1^)	1.33562	[1.31325, 1.36386]	0.97
ζ (–)	0.89949	[0.87125, 0.93150]	1.74
τ_a_ (s)	0.31687	[0.29951, 0.33878]	3.19
τ_b_ (s)	0.053639	[0.05237, 0.05490]	1.20
f_n_ = ω_n_/2π (Hz)	0.21257	[0.2090, 0.2171]	–
f_a_ = 1/2πτ_a_ (Hz)	0.50227	[0.4698, 0.5314]	–
f_b_ = 1/2πτ_b_ (Hz)	2.96717	[2.8988, 3.0389]	–

**Table 4:** Physical attribution of the identified zeros and poles. The counts are determined by the measurement; the assignment of poles to named circuit elements is a plausible reading, not an identified result.

Physical Element	Contribution	Identified Location
Pyroelectric current, i ∝ dT/dt	1 zero at the origin	–
AC-coupled conditioning stage	1 zero at the origin + 1 pole	–
Thermal and electrical relaxation of the element	complex pole pair	f_n_ = 0.2126 Hz, ζ = 0.899
Coupling/gain stage pole	1 real pole	f_a_ = 0.5023 Hz
Output low-pass filter	1 real pole	f_b_ = 2.9672 Hz

**Table 5:** Identifiability metrics of the full 40-point candidate pool, evaluated at the operating point identified in Section 3.

Quantity	Design A (Magnitude Only)	Design B (Magnitude + Phase)
Candidate conditions N	40	40
Number of parameters P	5	5
Size of J_s_	40 × 5	80 × 5
rank(J_s_)	5/5	5/5
σ_min_(J_s_)	1.8294 × 10^−1^	3.4839 × 10^−1^
σ_max_(J_s_)	1.3871 × 10^1^	1.4372 × 10^1^
κ(J_s_)	75.82	41.25
λ_min_(F_s_) (mV^−2^)	5.838 × 10^2^	2.117 × 10^3^
κ(F_s_)	5.749 × 10^3^	1.702 × 10^3^
log det F_s_	51.5365	56.4024

**Table 6:** Parameter sensitivity, weakest identifiable direction, and predicted versus observed precision for the dynamic block.

Parameter	Norm. Sens., A	Norm. Sens., B	Weakest Dir. (A)	CRLB, A (%)	CRLB, B (%)	Bootstrap (%)
K	0.556	0.528	−0.280	1.510	0.655	1.40
ω_n_	1.000	1.000	−0.199	1.003	0.630	0.97
ζ	0.243	0.290	−0.425	1.853	0.935	1.74
τ_a_	0.409	0.434	−0.804	3.345	1.824	3.19
τ_b_	0.147	0.223	+0.235	1.315	0.599	1.20

**Table 7:** Greedy D-optimal subsets of the 40-point candidate pool under the magnitude-only observation model. D-efficiency is referred to the full pool.

M	Rank(J_s_)	σ_min_(J_s_)	κ(J_s_)	λ_min_(F_s_) (mV^−2^)	Log Det F_s_	η^D^ (%)
5	5/5	6.970 × 10^−2^	63.79	8.473 × 10^1^	43.240	43.6
6	5/5	7.899 × 10^−2^	59.16	1.088 × 10^2^	44.131	47.7
8	5/5	1.133 × 10^−1^	49.70	2.238 × 10^2^	45.624	55.4
10	5/5	1.192 × 10^−1^	52.32	2.478 × 10^2^	46.688	61.6
12	5/5	1.367 × 10^−1^	50.93	3.260 × 10^2^	47.503	66.8
15	5/5	1.495 × 10^−1^	53.68	3.901 × 10^2^	48.410	73.1
20	5/5	1.608 × 10^−1^	58.15	4.511 × 10^2^	49.474	81.4
25	5/5	1.712 × 10^−1^	62.20	5.113 × 10^2^	50.214	87.6
30	5/5	1.753 × 10^−1^	66.96	5.360 × 10^2^	50.777	92.7
40	5/5	1.829 × 10^−1^	75.82	5.838 × 10^2^	51.537	100.0

**Table 10:** Screening design and measured response, laid out in two blocks of nine runs. Translations *Δ*x, *Δ*y, *Δ*z in mm, rotations *θ*_x_, *θ*_y_, *θ*_z_ in degrees, PSD in mV^2^. Runs 8–12 are replicated center points.

No.	Δx	Δy	Δz	θ_x_	θ_y_	θ_z_	PSD	No.	Δx	Δy	Δz	θ_x_	θ_y_	θ_z_	PSD
1	0	−1	2	−1	−0.1	−1	1521.89	10	0	0	0	0	0	0	465.84
2	2	−1	0	1	−0.1	1	9226.04	11	0	0	0	0	0	0	560.34
3	−2	0	−2	1	−0.1	−1	97,770.27	12	0	0	0	0	0	0	698.39
4	−2	1	2	0	−0.1	1	39,075.75	13	0	1	−2	1	0.1	1	355.67
5	2	1	−2	−1	−0.1	0	49,837.38	14	−2	1	0	−1	0.1	−1	7713.92
6	2	1	2	1	0	−1	9827.71	15	2	0	2	−1	0.1	1	142,435.33
7	−2	−1	−2	−1	0	1	419.52	16	2	−1	−2	0	0.1	−1	68,439.51
8	0	0	0	0	0	0	1380.56	17	−2	−1	2	1	0.1	0	32,616.54
9	0	0	0	0	0	0	603.09								

**Table 12:** Optimization-stage D-optimal design and measured response, referred to the origin relocated after screening, in two blocks of fourteen runs. Units as in Table 10.

No.	Δx	Δy	Δz	θ_x_	θ_y_	θ_z_	PSD	No.	Δx	Δy	Δz	θ_x_	θ_y_	θ_z_	PSD
1	−0.1	−0.1	0.1	−0.5	0.1	−0.5	6039.56	15	−0.1	0.1	−0.1	0	0	0	194.01
2	−0.1	0.1	0	0.5	0.1	−0.5	992.49	16	0	0	−0.1	0.5	0	0.5	468.97
3	0.1	0	0.1	0	0.1	−0.5	3932.58	17	0.1	0.1	−0.1	0.5	−0.1	−0.5	6549.58
4	0	0	−0.1	−0.5	0.1	−0.5	9326.88	18	−0.1	0	−0.1	−0.5	−0.1	−0.5	1116.12
5	0.1	−0.1	−0.1	0.5	0.1	0	361.03	19	−0.1	0	0.1	0.5	−0.1	−0.5	10,729.45
6	0.1	0.1	0	0	0.1	0.5	13,525.93	20	0.1	−0.1	0.1	−0.5	−0.1	−0.5	900.93
7	−0.1	−0.1	−0.1	−0.5	0.1	0.5	18,728.21	21	−0.1	−0.1	−0.1	0.5	−0.1	0	7585.32
8	−0.1	0	0.1	−0.5	0.1	0.5	18,274.69	22	0	0	0	0	−0.1	0	1361.03
9	0.1	−0.1	0.1	−0.5	0.1	0.5	25,451.94	23	0	0.1	−0.1	−0.5	−0.1	0.5	647.89
10	−0.1	−0.1	0.1	0.5	0.1	0.5	634.51	24	−0.1	0.1	0	0.5	−0.1	0.5	5640.62
11	0	0.1	0.1	−0.5	0	−0.5	1872.54	25	−0.1	−0.1	0.1	−0.5	−0.1	0.5	473.69
12	0	−0.1	0	0	0	−0.5	195.15	26	0.1	−0.1	0	0.5	−0.1	0.5	3438.41
13	0.1	0	0	−0.5	0	0	7134.43	27	0.1	−0.1	−0.1	0	−0.1	0.5	351.04
14	0	0.1	0.1	0.5	0	0	743.39	28	0.1	0.1	0.1	0	−0.1	0.5	659.55

**Table 13:** Alignment coordinates and measured response at each stage. Coordinates are cumulative relative to the initial alignment.

Stage	Δx (mm)	Δy (mm)	Δz (mm)	θ_x_ (deg)	θ_y_ (deg)	θ_z_ (deg)	PSD (mV^2^)
Initial	0	0	0	0	0	0	558.45
After screening	0	−0.21	1.02	0.60	0.02	0.10	300.28
After optimization	0	−0.31	0.92	0.70	0.02	−0.60	127.53

## Data Availability

The frequency-sweep measurements of Section 3, the design matrices and responses of Table 10 and Table 12, the identified model parameters and the D-optimal designs of Table 7, Table 8 and Table 9 are available from the corresponding author upon reasonable request. Figure 4, Figure 5, Figure 6, Figure 7, Figure 8 and Figure 9 are plotted directly from the frequency-sweep data; Figure 10 and Figure 11 are surfaces of the regression models of Table 11 fitted to the data of Table 10 and Table 12; Figure 2 is a computed illustration of Equations (15), (20) and (21) with the parameters listed in its caption; Figure 1, Figure 3 and Figure 12 are photographs and engineering drawings of the apparatus used in this work. No figure in this manuscript was generated synthetically or by an image-generation model.
